# Investigation of catalytic effect on carbon-carbon bond formation by Baylis-Hillman (BH) reaction between (2/3/4)-nitro-arylaldehyde and alkylacrylates and computational approaches through DFT functional

**DOI:** 10.1016/j.heliyon.2021.e08139

**Published:** 2021-10-15

**Authors:** Laila Arifun Nahar, Ajoy Kumer, Md Wahab Khan

**Affiliations:** Organic Research Laboratory, Department of Chemistry, Bangladesh University of Engineering and Technology (BUET), Dhaka, 1000, Bangladesh

**Keywords:** DABCO, Arylaldehyde, Alkylacrylates, Computational chemistry, Carbon-carbon bond and DFT

## Abstract

The 4-diazabicyclo[2. 2. 2] octane, DABCO, had widely established with gigantic and celestial applications on catalysis for carbon-carbon bond formation reactions. Thus, it has been employed to synthesize the alkyl 2-(hydroxyl (nitrophenyl) methyl)acrylate by the reaction of arylaldehydes and alkylacrylates under a mild condition with good yields. First of all, reaction was examined effect of various solvents, such as water, MeOH, Dioxane, DMSO, t-Butanol, DMF, Toluene, and THF and THF was the best solvent in term of yield. Next, the room temperature (R.T) was the optimized condition than 60 °C and 80 °C. The overall reaction progress was monitored in presence of DABCO, Et_3_N, C_2_H_5_ONa, C_4_H_9_OK, (CH_3_)_3_COK and pyridine catalysts with THF solvents at room temperatures, and calculated the amount for superior of catalyst. There was no product obtained in presence of catalysts, such as Et_3_N, C_2_H_5_ONa, C_4_H_9_OK, (CH_3_)_3_COK and pyridine. But in present of DABCO, this reaction has proceeded and monitored the concentration of catalyst and various temperature effects on reaction progress. In addition, the computational approaches for speculative investigation of solvents effect has employed for predicating and comparatively verified with the proposed reaction mechanism in presence of DABCO catalyst through the Density Functional Theory (DFT). The most acceptable tools for the thermodynamic had been illustrated to get the reaction kinetics by formation energy, entropy, enthalpy for reactant, product and transition state. Finally, the Gibbs free energy for reactions from the reactants and product has calculated to predict the occurring spontaneously possibility with and without solvents, and it is said that the reaction is spontaneously occurred through the water, DMSO, THF solvent although it is opposed fact without solvent even other solvents. It might be concluded that the optimization conditions of reaction are the THF solvent in presence of 20% DABCO catalyst at room temperature with high (about 90%) throughout 6–8 h where the 4- position of nitro group in arylaldehyde is the most preferable in case of time and yield.

## 1 Introduction

Morita-Baylis-Hillman (MBH) reaction, which was modified in 1972 by Anthony B. Baylis and Melville E. D. Hillman, illustrated the carbon-carbon single bond formation between activated alkene and carbon electrophilic compounds [1, 2] but it was originated by K. Morita in 1968 [3]. Although, there were various approaches, for example aldol reaction [4], Reformatsky reaction [5], Grignard reaction [6], Diels-Alder reaction [7, 8], Wittig reaction [9, 10], and Heck reaction [11, 12, 13] for erection of carbon-carbon bond(s) with well established literatures, but the MHB has taken the vast attention with gigantic significance in organocatalysis and heterocyclic synthesis [14], enantioselective synthesis of spirocyclohexenes [15], organocatalytic asymmetric transformations [16], and synthesis of isatin-derived ketimines [17]. In addition, it belongs to the functional groups generation and atom economy [18], use non-metal and low toxic materials for catalysis [19, 20], mild conditions and compatibility of multiple functional groups [21, 22], synthesis of natural products and drug molecules [23], Chiral binaphthyl-derived amine-thiourea [24], organomatallic and heterocyclic synthesis [25]. But the main drawback of the MBH reaction is the use of toxic metal as catalysts [26]. Regarding that point, Hill and Isaacs *et al.* 1990 [27], Fort *et al.* 1992 [27], Lewis acids [28], Lewis bases [29], E.P. Kristin *et al.* in 2005 [30] and so many scientist performed numerous researches during 1990–2005 on the catalytic process. In addition, a variety of 2-(1-hydroxyalkyl)-2-propenoic esters was prepared by using DABCO which has executed as acting a catalyst for coupling of aldehyde with methylacrylate [31], and Yong-Ling Shi and Min Shi proposed another similar concept for the DABCO-catalyzed reactions for salicyl *N*-tosylimine, with ethyl 2, 3-butadienoate and penta-3, 4-dien-2-one to produce the corresponding chromenes [31]. The stereoselective DABCO-catalyzed synthesis in (*E*)-α-ethynyl-α, β-unsaturated ester from allenyl acetates was accomplished by Yongsik Choe and Phil Ho Lee which opened a new dimension for application of DABCO for coupling reaction to form carbon-carbon bond without toxic metals [32]. The main fact of using DABCO as catalyst in MBH reaction is explained for its inexpensive and highly efficient catalytic activity, which has as well been developed for the cross-coupling of arylhalides with arylboronic acids [33]. The total synthesis of syributins using the BH adduct of 2, 3-*o*-isopropylidene-*R*-glyceraldehyde and ethylacrylate as starting material followed by ring closing metathesis of the acrylate derivative [34]. In recent time, the most applicable and popular research on BH which was published by Nyoung Kim *et al.* for the synthesis of 3, 4-disubstituted 2(*1H*)-quinolinones through intermolecular Friedel-Crafts reaction of *N*-arylamides of Baylis-Hillman adduct [35]. For having the vast applications of BH and MBH reactions, it has examined for the carbon-carbon unaccompanied bond construction between activated arylaldehydes and alkylacrylates in presence of DABCO catalyst which has as well modified by solvent and temperature effect; has been adding new dimension for synthesis of biologically significance molecules. Moreover, it must be noted for synthesis of alkyl 2-(hydroxyl (nitrophenyl) methyl)acrylate has no well-established methods for their synthesis. As a result, this study finds the sustainable method development with applying catalysts in temperature and solvents conditions that gives the assistance for introducing new method to get the high yield of methyl acrylate and its alkyl derivates as well as less time and low cost.

Besides the experimental evidences, the computational approaches have been applied in view of thermodynamics and chemical reactivity for supporting about reaction mechanism with solvent effect [36]. To illustrate the solvents effect for formation of the carbon-carbon bond formation to form methyl acrylate and its alkyl derivates from nitro arylaldehydes with different acrylates, the most accurate method of DFT has executed from material studio software [37], because it gives an extraordinary and accurate accomplishment for solving a potential problem of experimental work [38]. However, the accuracy of a theoretical method for some other problems may not entail precision for the crisis nearby. The potential energy, Gibbs free energy, HOMO- LUMO energy gap, chemical potential and entropy might be calculated for accurate explanation of catalytic and mechanistic studies of this reaction which can predict the possibility of occurring reaction with conditions. Some usual mechanistic steps, particularly carbon-carbon bond formation using solvent with catalyst, are sufficiently tractable in computational or quantum calculation [39, 40, 41, 42, 43, 44]. Finally, this theoretical hypothesis has used to understand by computational mechanistic results, specifically thermodynamic parameter and chemical reactivity of reactant, product and transition state to get accurate result which is compatible with experimental studies through the solvent and DABCO catalyst.

## 2 Experimental materials and methods

### 2.1 Methods and materials

IR spectra were recorded on a Shimadzu FTIR spectrophotometer and UV spectra were recorded in dry CHCl_3_ with Shimadzu visible spectrophotometer. ^1^H NMR and ^13^C NMR spectra were recorded on a Bruker DPX-400 spectrophotometer (400 MHz) using tetramethylsilane as internal reference. Analytical thin-layer chromatography (TLC) was performed on pre-coated silica gel 60 F-254 (E. Merck). Column chromatography was performed on silica gel (60–120 mesh). DABCO (1, 4-Diazabicyclo [2. 2. 2] octane), acrylates, aldehydes and other reagents were purchased from E. Merck (Germany) and Fluka (Switzerland).

### 2.2 General experimental methods

**Typical reaction procedure for the preparation of alkyl 2-(Hydroxy (nitrophenyl) methyl) acrylate 6-11**

Alkyl 2-(hydroxyl (nitrophenyl) methyl) acrylate **(6–11)** were prepared from the reaction of nitro arylaldehydes with different acrylates in the presence of DABCO at room temperature. To a stirred solution of methyl acrylate (500 mg, 5.81 mmol) and DABCO (137 mg, 1.16 mmol, 20 mol %) in tetrahydrofuran (5 ml) at 28–30 °C, nitrobenzaldehyde (1932 mg, 12.78 mmol) was added slowly. Then the mixture was stirred for 6 h. The progress of the reaction was monitored by TLC. Then the solvent of the reaction mixture was removed by distillation and extracted by CHCl_3_ solution (3 × 50 mL), the organic layer was washed with distilled water (3 × 50 mL) and dried over anhydrous Na_2_SO_4_. The crude product obtained after evaporation of the solvent was purified by chromatography on a column of silica gel (60–120 mesh) with ethyl acetate in n-hexane to yield light yellowish solid pure compound **6**. The spots were visualized with UV light, the Rf value of TLC was at 0.6–0.65 which is acceptable to predict the monitoring reaction progress.

### 2.3 Characterization by spectral data of synthesized compounds

All the synthesized compounds were characterized by their satisfactory spectroscopic (UV, IR, ^11^H NMR, ^13^C NMR) and elemental analysis. The formation of the coupling products was established on the basis of the spectroscopic data observations. For the characterization by ^1^H NMR spectrum of alkyl 2-(hydroxy (nitrophenyl) methyl) acrylates (7) was accounted for the chemical shift at 8.11 (d, 2H, Ar–H), 7.52 (d, 2H, Ar–H), 6.35 (s,1H, 1-ethylene), 5.90 (s,1H, 1-ethylene), 5.61 (s, 1H, methine), 3.68 (s, 3H, CH_3_). There was not presence any chemical shift at 9.45 ppm which was confirmed the absence of peak for –CHO group and in case of the –CH = CH_2_ group of acrylates, the original peak of that ethylene group was shown at 6.05 ppm considering its conversion. However, the absence chemical shift at original peak region of –CHO and –CH = CH_2_ groups pointed out the conversion into aldehyde group and ethylene group in alkyl 2-(hydroxy (nitrophenyl) methyl) acrylates compound (6). For giving the strongest evidence for structural conversion, the ^13^C NMR spectrum of alkyl 2-(hydroxy (nitrophenyl) methyl) acrylates (6) was accounted for the chemical shift at 166.33 (CO), 148.90 (Ar–C), 147.32 (Ar–C), 141.17 (1-ethylene), 127.459 (Ar-CH), 127.03 (1ethylene), 123.53 (Ar-CH), 72.23 (C-methine), 52.14 (CH_3_). For the fact of FTIR, the strongest peaks for stretching at about 3508.63 cm^−1^ (O–H) asymmetry 3310.37 cm^−1^ (O–H) symmetry make available the existence of C–C new bond formation at this point, on top of another three peaks at 1700 cm^−1^ (C–O) asymmetry, 1670 cm^−1^ (C–O) symmetry prove the existence of carboxylate ion, and 1350 cm^−1^ for the –NO_2_ group. Finally, the FTIR spectrum, ^13^C NMR spectrum, and ^1^H NMR spectrum of compound 7, 8, 9, 10, and 11, were similar to compound 6 and confirmed their functional groups. Withal, the UV spectrum for compound 6 shows the similar absorption at about 394.50, 271.50, 218.50 nm wave-length which is almost similar to other compounds (7, 8, 9, 10 and 11).

**Methyl 2-[hydroxyl (4-nitrophenyl) methyl] acrylate 6**

Light Yellowish solid; mp 72–73 °C, Yield: 92%, 1.266 g

IR: ν_max_ (KBr) 3508.37 (O–H), 2852–3108 (C–H), 1700 (C–O), 1601.93 (-O-), 1350 (NO_2_), 700-1000 cm^−1^.

UV (CHCl_3_): λ_**max**_ 394.50, 271.50, 218.50 nm.

^1^HNMR (400 MHz, CDCl_3_) δ (ppm) 8.11 (d, 2H, *J* = 8.4 Hz, Ar), 7.52 (d, 2H, *J* = 8.8 Hz, Ar), 6.35 (s, 1H, 1-ethylene), 5.90 (s, 1H,1-ethylene), 5.61 (s, 1H, methine), 3.68 (s, 3H, CH_3_).

^13^C NMR (100 MHz, CDCl_3_) δ (ppm) 166.33 (CO), 148.90 (Ar–C), 147.32 (Ar–C), 141.17 (1-ethylene-CO), 127.46 (Ar-CH), 127.03 (1-ethylene), 123.53 (Ar-CH), 72.24 (C-methine), 52.14 (CH_3_).

Anal. Calcd for C_11_H_11_NO_5_: C, 55.70; H, 4.67; N, 5.90. Found: C, 55.85; H, 4.82; N, 6.10.

**Ethyl 2-[hydroxyl (4-nitrophenyl) methyl] acrylate 7**

Light yellowish liquid, Yield: 90%, 1.129 g

IR: ν_max_ (KBr) 3500.92 (O–H), 2986–3134 (C–H), 1710 (C–O), 1610 (-O-), 1350 (NO_2_), 700–1000, cm^−1^.

UV (CHCl_3_): **λ**_**max**_ 336.00, 273.00, 218.80, 214.40, 204.60 nm.

^1^H NMR (400 MHz, CDCl_3_) δ (ppm) 8.16 (d, 2H, *J* = 7.08 Hz, Ar), 7.54 (d, 2H, *J* = 7.55 Hz, Ar), 6.37 (s, 1H, 1-ethylene), 5.85 (s, 1H, 1-ethylene), 5.60 (s, 1H, methine), 4.134 (q, 2H, *J* = 7.72, 7.71 Hz, –OCH_2_)), 3.48 (s, 1H, OH), 1.23 (t, 3H, *J* = 5.57, 5.58 Hz, CH_3_).

^13^C NMR (100 MHz, CDCl_3_) δ (ppm) 165.93 (CO), 148.77 (Ar–C), 147.35 (Ar–C), 141.20 (1-ethylene-CO), 127.34 (Ar-CH), 126.96 (1-ethylene), 123.54 (Ar-CH), 72.61 (C-methine), 61.29 (-OCH_2_), 13.98 (CH_3_).

DEPT: 127.34 (Ar-CH), 126.96 (1-ethylene), 123.50 (Ar-CH), 72.34 (C-methine), 61.29 (-OCH_2_), 13.98 (CH_3_).

**Methyl 2-[hydroxy (3-nitrophenyl) methyl] acrylate 8**

Colourless liquid; Yield: 83%, 1.143 g

IR: ν_max_ (KBr) 3476.81(O–H), 2987.84 (C–H), 1709.95 (C–O), 1531.53 (–CO–), 1352.14 (NO_2_), 700-1000 cm^−1^.

UV (CHCl_3_): **λ**_**max**_ 676.50, 263.50, 224.50 nm.

^1^H NMR (400 MHz, CDCl_3_) δ (ppm) 8.26 (s, 1H, Ar), 8.14 (d, 1H, *J* = 7.2 Hz, Ar), 7.74 (d, 1H, *J* = 7.6 Hz, Ar), 7.52 (t, 1H, *J* = 8.0 Hz, Ar), 6.42 (s, 1H, 1-ethylene), 5.93 (s, 1H, 1-ethylene), 5.65 (s, 1H, methine), 3.75 (s, 3H, CH_3_).

^13^C NMR (100 MHz, CDCl_3_) δ (ppm) 166.39 (CO), 148.37 (Ar–C), 143.67 (Ar–C), 141.04 (1-ethylene), 132.68 (Ar-CH), 129.36 (Ar-CH), 127.26 (Ar-CH), 122.77 (Ar-CH), 121.57 (1-ethylene), 72.56 (C-methine), 52.19 (CH_3_).

Anal. Calcd for C_11_H_11_NO_5_: C, 55.70; H, 4.67; N, 5.90. Found: C, 55.87; H, 4.78; N, 6.08.

**Ethyl 2-[hydroxy (3-nitrophenyl)methyl] acrylate 9**

Colourless liquid; Yield: 70%, 0.878 g

IR: ν_max_ (KBr) 3489.34 (O–H), 2956.01 (C–H), 1712.85 (C–O), 1536.53 (–CO–), 1350 (NO_2_), 700-1000 cm^−1^.

UV (CHCl_3_): **λ**_**max**_ 652.50, 264.00, 230.00 nm.

^1^H MR (400 MHz, CDCl_3_) δ (ppm) 8.27 (s, 1H, Ar), 8.14 (d, 1H, *J* = 8.0 Hz, Ar), 7.75 (d, 1H, *J* = 8.0 Hz, Ar), 7.51 (t, 1H, *J* = 8.0 Hz, Ar), 6.42 (s, 1H, 1-ethylene), 5.91 (s, 1H, 1-ethylene), 5.64 (s, 1H, methine), 4.17 (q, 2H, *J* = 7.2, 6.8 Hz, O–CH_2_), 3.76 (s, 1H, OH), 1.26 (t, 3H, *J* = 7.2, 6.8 Hz, CH_3_).

^13^C NMR (100 MHz, CDCl_3_) δ (ppm) 165.95 (CO), 148.33 (Ar–C), 143.67 (Ar–C), 141.28 (1-ethylene), 132.67 (Ar-CH), 129.36 (Ar-CH), 126.95 (Ar-CH), 122.72 (Ar-CH), 121.57 (1-ethylene), 72.56 (C-methine), 61.30 (O–CH_2_), 14.02 (CH_3_).

Anal. Calcd for C_12_H_13_NO_5_: C, 57.37; H, 5.22; N, 5.58. Found: C, 57.52; H, 5.45; N, 5.71.

**Methyl2-[hydroxy(2-nitrophenyl)methyl] acrylate 10**

Light yellowish liquid; Yield: 80%, 1.102 g

IR: ν_max_ (KBr) 3446.91(O–H), 2956.01 (C–H), 1718.63 (C–O), 1532.50 (–CO–), 1352.14 (NO_2_), 600-1000 cm^−1^.

UV (CHCl_3_): **λ**_**max**_ 257.40, 214.20 nm.

^1^H NMR (400 MHz, CDCl_3_) δ (ppm) 7.95 (d, 1H, *J* = 8 Hz, Ar), 7.76 (d, 1H, *J* = 7.6 Hz, Ar), 7.64 (t, 1H, *J* = 7.6 Hz, Ar), 7.46 (t, 1H, *J* = 7.2, 8.0 Hz, Ar), 6.38 (s, 1 H, 1-ethylene), 6.22 (s, 1H, 1-ethylene), 5.74 (s, 1H, methine), 3.75 (s, 3H, CH_3_).

^13^C NMR (100 MHz, CDCl_3_), δ (ppm) 166.43 (CO), 148.36 (Ar–C), 140.78 (Ar–C), 136.11 (1-ethylene), 133.45 (Ar-CH), 128.91 (Ar-CH), 128.71 (Ar-CH), 126.47 (Ar–H), 124.58 (1-ethylene), 67.70 (C-methine), 52.17 (CH_3_).

Anal. Calcd for C_11_H_11_NO_5_: C, 55.70; H, 4.67; N, 5.90. Found: C, 55.62; H, 4.73; N, 5.78.

**Ethyl 2-[hydroxyl (2-nitrophenyl) methyl] acrylate 11**

Light yellowish liquid; Yield: 84%, 1.054 g

IR: ν_max_ (KBr) 3518.28 (O–H), 2986.87 (C–H), 1719.60 (C–O), 1532.50 (–CO–), 1332.50 (NO_2_), 600-1000 cm^−1^.

UV (CHCl_3_): **λ**_**max**_ 355.60, 335.60, 256.00, 211.80 nm.

^1^H NMR (400 MHz, CDCl_3_) δ (ppm) 7.97 (d, 1H, *J* = 8 Hz, Ar), 7.75 (d, 1H, *J* = 7.2 Hz, Ar), 7.64 (t, 1H, *J* = 7.6 Hz, Ar), 7.46 (t, 1H, *J* = 8.4, 8.0 Hz, Ar), 6.39 (s, 1H, 1-ethylene), 6.20 (s, 1H, 1-ethylene), 5.75 (s, 1H, methine), 4.15 (Q, 2H, *J* = 4.6, 7.2 Hz, OCH_2_), 2.86 (s, 1H, OH), 1.21 (t, 3H, *J* = 7.2 Hz, CH_3_).

^13^C NMR (100 MHz, CDCl_3_), δ (ppm) 165.97 (CO), 148.41 (Ar–C), 140.90 (Ar–C), 136.22 (1-ethylene), 133.49 (Ar-CH), 128.93 (Ar-CH), 128.70 (Ar-CH), 126.29 (Ar–H), 124.57 (1-ethylene), 67.75 (C-methine), 61.20 (OCH_2_), 13.99 (CH_3_).

Anal. Calcd for C_12_H_13_NO_5_: C, 57.37; H, 5.22; N, 5.58. Found: C, 57.54; H, 5.38; N, 5.75.

It is reported here that the coupling between alkylacrylates with arylaldehydes were accomplished in presence of DABCO in good yield% (All of spectrum data was attached in supplementary file in Figure-S2, Table S1).

### 2.4 Computational details

To kick off, the development of chemical reaction mechanism through the solvent effect, the DFT functional has been employed from DMol^3^ code of material studio 8.0. To perform this work, the B3LYP functional and DND basis set was used. In this case, the reactants and products were used to form the transition state. Next, the reactants, products and transition complexes were simulated using the B3LYP functional and DND basis set and recorded to the heat of formation, entropy, enthalpy and Gibbs free energy. Secondly, the evaluate the solvent effect, the reactant and product were also simulated using the same method and condition, and Water, MeOH, Dioxane, DMSO, t-Butanol, DMF, Toluene, and THF solvent were executed from the DFT methods to calculate the Gibbs free energy and heat of formation. On the other hand, to calculate the quantum properties, structural stability, the B3LYP functional and DND basis set from the DMol^3^ code of material studio 8.0. The HOMO, LUMO and Electrostatic potential 3D map were optimized and analyzed for illustrate the chemical descriptors. At last for calculating the stereocnetre of optimized molecules, the Gaussian 16 software packet was used for determination the stereoisomer for the synthesized molecules [45, 46]. In this case, the B3LYP function with 6-311G basis set from DFT functional method was performed for optimization. After optimization the stereoisomer of optimized molecules was determined to give specification of R and S center.

## 3 Results and discussions

### 3.1 Chemistry

The aldehydes and acrylates were used for synthesis of alkyl 2-(hydroxyl (nitrophenyl) methyl) acrylate and its derivatives which were characterized by ^1^H NMR, ^13^C NMR, UV spectra and FTIR (attached the spectral diagram in supplementary file S2). The reaction progress was monitored in presence of various solvents medium to get the optimized products with high yield. No reaction was ensued without DABCO catalyst only using solvent. After adding solvent, this reaction was persisted the product where the 4-nitro arylaldehyde shows the highest yield rather than 3-nitro arylaldehyde and 2-nitro arylaldehyde, so that it must be concluded that the nitro group at 4-position of aryl ring was highly preferable for occurring this reaction. The main reason may be explained as that the higher distance of the nitro group would stay minimum static hindered for reactant during the reaction progress. On the other hand, another imperative memorandum is mentioned that larger alkyl chain of acrylate is required more time and low yield. For justification of reaction mechanism, the 1^st^ order is possible in presence of DABCO catalyst.

### 3.2 Proposed mechanism and effect of solvents as well as temperature

Effects of solvent, concentration of aldehydes and acrylates, and DABCO at different temperatures were premeditated, and it was recorded that the yield of the expected product depended on the nature of the base, temperature and solvents in present of DABCO catalyst. To kick off, the solvent effects on the reaction was examined using a variety of solvents, such as 1, 4-dioxane, tetrahydrofuran (THF), dimethylsulfoxide (DMSO), dimethylformide (DMF), dichloromethane (DCM), toluene, acetonitrile, *t*-butanol and methanol. Dioxane, DCM, *t*-butanol and DMSO. It is reported here alkyl 2-(hydrxy (nitrophenyl) methyl) acrylate **6–11** was obtained by treating 1mol of acrylate **(4–5)** with 2.2 mol of arylaldehyde **(1–3)** in the presence of DABCO (20 mol%) in anhydrous THF at room temperature for 6 h in **pathway-3 and S1**. The product was purified by chromatography on a column with ethyl acetate in n-hexane to yield pure compound **6** of 90% yield in **optimization table, Entry-1**. As higher yield of compound **6** was observed by using DABCO 20 mol%, Tetrahydrofuran (THF) solvent at R.T. for staring time 6 h are shown in optimization Table 1. From this table we observed MeOH was better solvent than others in this Tables 1, 2, 3, 4 Dioxan, Dichloromethane (DCM), t-Butanol and dimethyl sulfoxide (DMSO) were found to be poor solvent due to less solubility obtaining 50% yield in **Entry-5**. DMF and toluene were found to be the moderately good solvent to obtain 60% yield in **Entry-6**. When the reaction was carried out in neat conditions, the product was obtained in lower yield. THF was originated to be the best solvent accounting on basis of yields of product.

**Table 1:** Optimization for preparation of methyl 2-(hydrxy (4-nitrophenyl) methyl) acrylate **6**.

Entry/Serial no	Base catalyst (mol%)	Solvent (20mL)	Temp. (°C)	Time (hr.)	Yield (%)
1	DABCO (20)	THF	R.T	6	90
2	DABCO (10)	THF	R.T, 60, 80	6 to 24	70
3	DABCO (100)	THF	R.T, 60, 80	6 to 24	60
4	DABCO (20)	MeOH	R.T	6	80
5	DABCO (20)	Dioxane or DCM or DMSO or t-Butanol.	R.T	6	50
6	DABCO (20)	DMF or Toluene	R.T	6	60
7	Et_3_N or C_2_H_5_ONa or C_4_N_9_OK or (CH_3_)_3_COK or Pyridine.	THF	R.T	6 to 24	Nil

**Table 2:** Data for proposed 1^st^ order reaction mechanism.

		Enthalpy ΔH, cal/mol	Tem pΔTK	Entropy, ΔS, cal/K/mol	TΔS	Free energy, ΔG, cal/mol	ΔE, Total energy, eV	Heat of formation Kcal/mol
Step-I	R-1	8222.714	298	119.723	35677.45	-27454.74	-2575.781	98.380
	TS-1	13045.71	298	164.990	19167.02	-6121.29	-2575.414	106.829
	P1	7249.348	298	99.191	29558.91	-22309.57	-2576.001	93.298
Step-II	R2	13116.06	298	140.841	41970.61	-28854.55	-4692.357	389.909
	TS-2	15403.19	298	179.592	53518.41	-38115.22	-4710.573	-30.180
	P2	12355.91	298	133.763	39861.37	-27505.46	-4692.353	389.984
Step-III	R3	12355.91	298	133.763	39861.37	-27505.46	-4692.353	389.984
	TS3	10677.66	298	132.387	39451.32	-28773.66	-3361.068	8.163
	P3	9156.11	298	113.697	33881.70	-24725.59	-3350.089	261.343
**THF solvent**								
Step-I	R1	8177.648	298	118.455	35299.59	-27121.94	-2576.137	90.174
	TS1	13101.232	298	165.061	49188.17	-47876.93	-2575.657	101.245
	P1	7278.158	298	99.466	29640.86	-22362.70	-2576.529	81.121
Step-II	R2	13202.450	298	141.864	42275.47	-29073.02	-4693.128	372.120
	TS2	15532.802	298	180.326	53736.85	-38204.04	-4711.093	-42.165
	P2	12311.950	298	133.632	39822.33	-27510.38	-4693.545	362.50
Step-III	R3	12311.950	298	133.632	39822.33	-27510.38	-4693.545	362.50
	TS3	10816.015	298	135.184	40284.83	-29468.81	-3361.434	-0.2828
	P3	9094.142	298	112.024	33383.15	-24289.00	-3350.585	249.9055

**Table 3:** Data for proposed 2^nd^ order reaction mechanism.

Steps		Enthalpy ΔH, cal/mol	Temp ΔT, K	Entropy, ΔS, cal/K/mol	TΔS	Free energy, ΔG, cal/mol	ΔE, Total energy, eV	Heat of formation Kcal/mol
Step-III B	R-3b	17577.911	298	174.503	52001.89	-34423.97	-5974.135	768.627
	TS-3b	7474.837	298	104.802	31230.99	-23756.15	-5961.955	734.590
	P-3b	15968.143	298	154.285	45976.93	-30008.78	-5972.744	485.788
Step-IV B	R-4b	15931.941	298	157.523	46941.85	-31009.90	-5945.254	1434.642
	TS-4b	15931.941	298	157.523	46941.85	-31009.90	-5947.540	1435.042
	P-4b	15297.826	298	154.087	45917.92	-30620.09	-5943.904	1465.760
Step-V B	R-5b	16252.486	298	161.316	48072.16	-31819.67	-5987.921	450.715
	TS-5b	16173.878	298	161.680	48180.64	-32006.76	-5988.921	451.001
	P-5b	4969.022	298	91.466	27256.86	-22287.64	-4625.448	473.168
**THF solvent**								
Step-III B	R-3b	17600.761	298	176.575	52619.64	-35018.87	-5975.445	738.413
	TS-3b	12415.732	298	134.143	39974.61	-27558.87	-5963.494	699.102
	P3b	13517.206	298	139.31	41514.38	-27997.17	-5974.1188	454.098
Step-IV B	R-4b	15970.438	298	159.161	47429.97	-31459.53	-5946.694	1421.493
	TS-4b	15964.565	298	159.159	47429.38	-31464.82	-5946.694	1401.430
	P-4b	15270.210	298	154.296	15980.20	-709.99	-5945.485	1429.312
Step-V B	R-5b	16288.847	298	161.681	48180.93	-31892.08	-5989.070	424.222
	TS-5b	16148.740	298	160.436	47809.92	-31661.18	-5989.070	424.222
	P-5b	4858.325	298	91.457	27254.18	-22395.85	-4625.794	465.186

**Table 4:** Data of the Gibbs free energy for reaction for proposed 1^st^ order reaction mechanism.

	Different stages	Free energy, ΔG, cal/mol	Free energy, ΔG, eV	ΔE, Total energy, eV	E_tcorr_ = ΔG +ΔE	The Gibbs free energy for reaction	Occurring possibility
Step-I	R- I	-27454.74	-1.190	-2575.781	-2576.97	+0.01	Not spontaneously
	TS-I	-6121.29	-0.265	-2575.414	-2575.67		
	P-I	-22309.57	-0.967	-2576.001	-2576.96		
Step-II	R-II	-28854.55	-1.251	-4692.357	-4693.60	+.06	Not spontaneously
	TS- II	-38115.22	-1.652	-4710.573	-4712.22		
	P- II	-27505.46	-1.192	-4692.353	-4693.54		
Step-III	R- III	-27505.46	-1.192	-4692.353	-4693.54	+1342.38	Not spontaneously
	TS- III	-28773.66	-1.247	-3361.068	-3362.31		
	P- III	-24725.59	-1.072	-3350.089	-3351.16		
**THF solvent**							
Step-I	R- I	-27121.94	-1.176	-2576.137	-2577.31	-0.18	Spontaneously occurred
	TS-I	-47876.93	-2.0761	-2575.657	-2577.71		
	P-I	-22362.70	-0.969	-2576.529	-2577.49		
Step-I	R-II	-29073.02	-1.260	-4693.128	-4694.38	-0.35	Spontaneously occurred
	TS- II	-38204.04	-1.656	-4711.093	-4712.74		
	P- II	-27510.38	-1.192	-4693.545	-4694.73		
Step-III	R- III	-27510.38	-1.192	-4693.545	-4694.73	+1343.10	Not Spontaneously occurred
	TS- III	-29468.81	-1.277	-3361.434	-3362.71		
	P- III	-24289.00	-1.053	-3350.585	-3351.63		

To optimize the reaction conditions, the reaction was observed at different temperature such as 80 °C, 60 °C and at room temperature. Room temperature was found to be the best condition for THF solvent. The yields got during heating are lower because of the parasitic reaction that can take place under these conditions between BH adducts and methanol which gives methylated ethers. The reaction was examined by employing different base catalyst, such as DABCO, Et_3_N, C_2_H_5_ONa, C_4_H_9_OK, (CH_3_)_3_COK and pyridine. When other base catalyst was employed without DABCO no product was observed after 6–24 h. The strong base (EtONa, ^t^BUOK etc) does not facilitate this reaction and it is likely to witness trans-Esterification of the acrylic esters. But when DABCO was employed as catalyst, the product was obtained in 92% after only 6 h. So DABCO is the best base catalyst for this reaction. Then it was examined the reaction by employing a different amount of DABCO such as 10 mol%, 20 mol% and 100 mol%. Increasing the amount of DABCO from 10 to 100 mol%, did not increase the yield% of compound **6**. The best result (90% yield) was got in case of 20 mol% DABCO catalyst. The examination of reaction time revealed that 6 h was found to be required to complete the reaction in presence of THF at room temperature. Therefore, the best reaction conditions were found to carry out the BH reaction with 2.2 equivalent of arylaldehyde **1–3** with 1 equivalent of acrylate **4–5** in presence of catalyst DABCO only (20 mol%) at room temperature in THF for 6hr. The coupling reactions of tolualdehyde, anisaldehyde, benzaldehyde, *p*-chlorobenzaldehyde, acetaldehyde, cinnamaldehyde, 4-formalbenzoic acid and 2-formalbenzoic acid with acrylates were performed under the same conditions, but no desired compound was afforded. On the other hand, the coupling reaction of methyl methacrylate, 2-hydroxy ethylmethacrylate and vinylacetate with arylaldehydes under the same conditions yielded no products. The BH reaction does not work with methylmethacrylates and its derivatives because it requires the presence of a vinyl proton in the alpha position of the ester group. It was revealed that the methyl group at the vinyl position hindered the coupling reaction at the same position. All the synthesized compounds **6–11** were characterized by IR, UV, ^1^HNMR, ^13^CNMR, and elemental analysis. The analytical data of compound **6** was found to be compatible with literature data [47].

**Figure 1:**
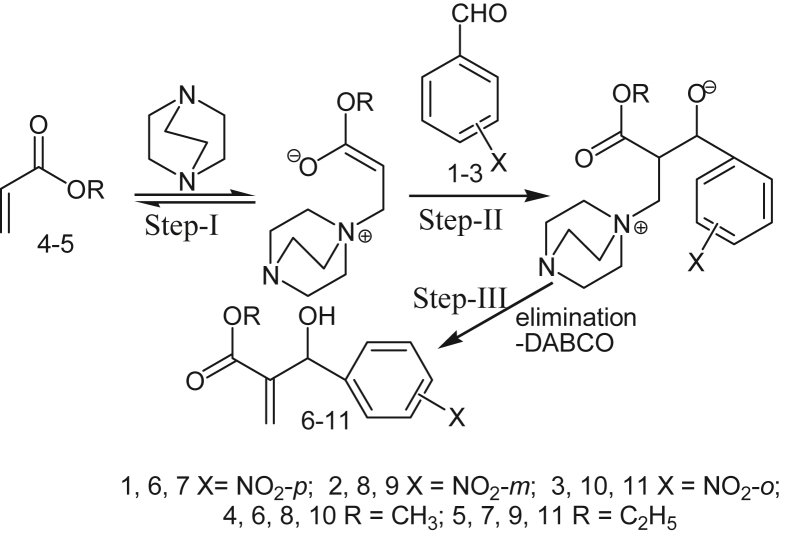
Proposed Reaction pathway-1 for 1^st^ order reaction.

**Figure 2:**
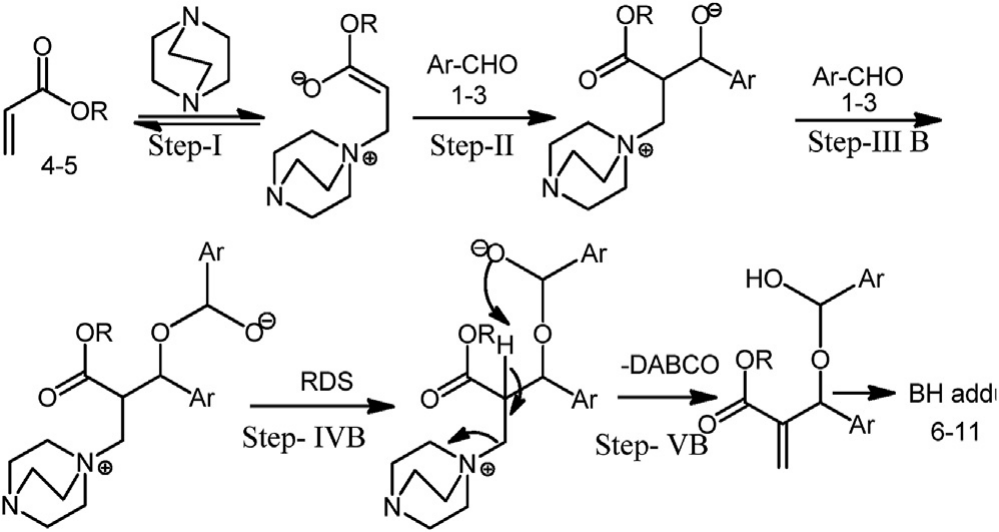
Proposed Reaction pathway-2 for 2^nd^order reaction.

Although the detailed mechanism of the coupling reactions is yet to be clarified, it can be perceived that the reactions proceed according to Baylis-Hillman mechanism. It has undoubtedly transparent that the presence of DABCO as base catalyst was indispensable condition for the coupling reaction between aldehyde and acrylate. The plausible mechanism is shown in the Figures 1 and 2 for 1^st^ order and 2^nd^ order reaction, respectively. However, Mc Quade et al; 2005; suggested a new mechanism based on the reaction rate data collected in aprotic solvents for BH reaction, that it is second order reaction in aldehyde with no catalyst but follows the first order in presence of DABCO catalyst [30]. For justification of proposed reaction mechanism, the computational tools were used.

### 3.3 Computational evidences for proposed mechanism and effect of solvents

**Figure 3:**
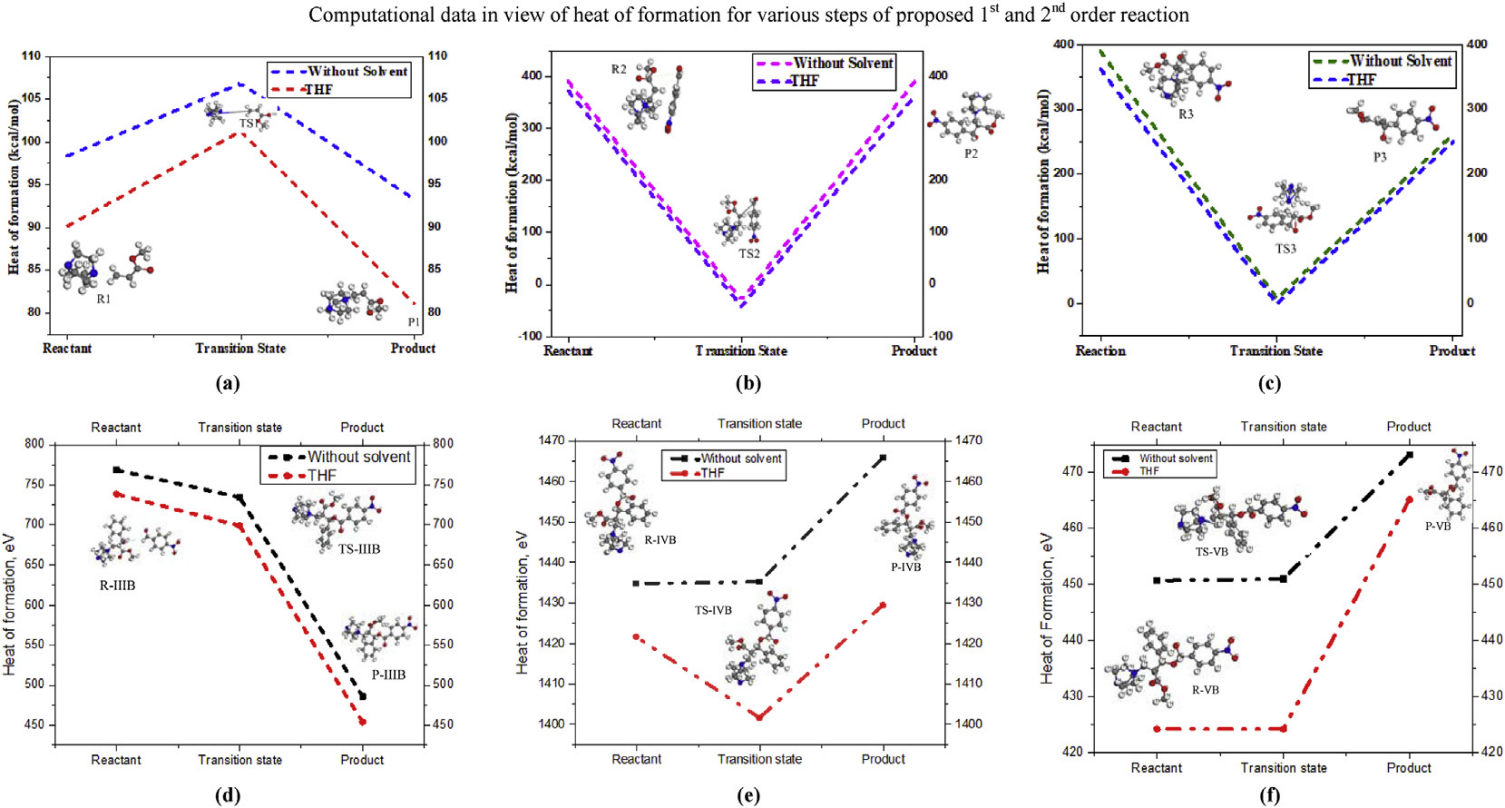
a) Step-I for 1^st^ and 2^nd^ order reaction, b) Step-II for 1^st^ and 2^nd^ order reaction, c) Step-III for 1^st^ order reaction, d) Step-IIIB for 2^nd^ order reaction, e) Step-IVB for 2^nd^ order reaction, f) Step-VB for 2^nd^ order reaction for Heat of formation for all steps of 1^st^ and 2^nd^ order reaction mechanism.

According to the theory by Mc Quade and co-workers for reaction mechanism of Baylis-Hillman reaction in aprotic solvents, the rate of reaction is completely depending through the 1^st^ order reaction kinetics in DABCO catalyst with acrylate reactant. For giving the strong evidence through the computational data, the formation energy for each stage of this proposed mechanism has illustrated in Figure 3 for both of solvent and without solvent medium.

First, it might be revealed for 1^st^ order reaction that the heat of formation is lower in THF solvent than without solvents. However, it could be concluded in such way that this reaction is easily occurred in solvent medium which is the best supportive for experimental evidence of solvent effect. Second, the formation energy of reactant is lower than transition state but higher than product in step-I, II and III steps, shown in Figure 3, which can be said the occurring possibility of these steps.

On the other hand, the formation energy for 2^nd^ order reaction, showing in Figure 3, of reactant, transition state and product are given this Figure 3 where the formation energy of all components without solvent is higher than THF solvent medium. In case of step IIIB, the formation energy of reactant is higher than transition state and product, but it is opposite for IVB and VB step. It can be said that the reaction does not easily follow the 2^nd^ order reaction as 1^st^ order reaction. Therefore, the proposed reaction pathway 1 is highly reliable and empirical than reaction pathway 2.

### 3.4 Computational approaches for reaction mechanism in view of Gibbs free energy

The change in the Gibbs free energy for a reaction (ΔG°) is the difference between the free energy of the products and the free energy of the reactants. When the reactants have higher free energy than the products (ΔG°_rxn_ <0), the reaction is called exothermic reaction, it opposite event is called endothermic reaction.

Free energy or, more appropriately, Gibbs free energy (after the inventor of the concept, J. Willard Gibbs) is a composite thermodynamic concept involving both enthalpy and entropy. It is given by *G* = *H* – *TS*. If a slight change occurs in a system kept at constant temperature, the resulting Gibbs free energy change is given by

Δ*G* = Δ*H* – *T*Δ*S*

To explain the reaction pathway, the Gibbs free energy is a tool derived from thermodynamic study calculating both of experimental and theoretical chemistry or computational chemistry. For different reaction pathways occurring possibility depends on Gibbs free energy, lower Gibbs free energy indicates the higher occurring possibility. To say more, if change of Gibbs free energy for a reaction is negative, then it's spontaneous. If change of Gibbs free energy is positive, meaning the non-spontaneous.

In order to explain the reaction mechanism with the computational chemistry, the thermodynamics of reactant, transition state and product have been considered in this study by the DMol^3^ code of the material studio 8.0 [48,49]. At each step of the reaction mechanism for the reaction and product, Gibbs free energy and formation energy has determined. Tables 2, 3 show that the steps I and II occur spontaneously, but the subsequent steps are unlikely to occur spontaneously. With the first order reaction, it is seen that the last one is not happening spontaneously. However, when the reaction mechanism THF solvent is used, the Gibbs free energy value is reduced, and the reaction occurs spontaneously. Again, the kinetics of the reaction depends on the first and second steps. Thus, it can be said that the reaction through computational chemistry by thermodynamics calculations supports the reaction for 1^st^ order and it is more convincing to solvent, THF.

According to the Gibbs free energy for reaction, it can be calculated by following equation for each step of predicted reaction mechanism

Δ*G reaction* = E_Tcorr_ (product)- E_Tcorr_ (reactant)

Here, E_Tcorr_ (product) and E_Tcorr_ (reactant) are the total energy of product and reactant, respectively which is equivalent to the sum of total energy and free energy.

From the above equation, if the calculated value will be positive, then this reaction does not occur in spontaneously at room temperature but the negative value shows its opposite phenomenon. From the Tables 4 and 5, we might find that the reaction Figure 1 (1^st^ order reaction) is not spontaneous without solvent medium. In solvent medium, it has spontaneously occurred and Gibbs free energy for reaction turns in negative values, and it indicates the occurring spontaneous possibility of reaction. It might be said that there have positive effect of solvent on reaction progress. In case of 2^nd^ order reaction (Figure 2), it is not spontaneous in both of solvent or without solvent.

**Table 5:** Data of the Gibbs free energy for reaction for proposed 2^nd^ order reaction mechanism.

Steps		Free energy, ΔG, cal/mol	Free energy, ΔG, eV	ΔE, Total energy, eV	E_tcorr_ = ΔG +ΔE, eV	The Gibbs free energy for reaction	Occurring possibility
Step-III B	R- IIIB	-34423.97	-1.492	-5974.135	-5975.627	+1.582	Not Spontaneously occurred
	TS- IIIB	-23756.15	-1.030	-5961.955	-5963.025		
	P- IIIB	-30008.78	-1.301	-5972.744	-5974.045		
Step-IV B	R-IVB	-31009.90	-1.344	-5945.254	-5946.598	+1.367	Not Spontaneously occurred
	TS- IVB	-31009.90	-1.344	-5947.540	-5948.884		
	P- IVB	-30620.09	-1.327	-5943.904	-5945.231		
Step-V B	R- VB	-31819.67	-1.379	-5987.921	-5989.300	+1362.886	Not Spontaneously occurred
	TS- VB	-32006.76	-1.387	-5988.921	-5990.308		
	P- VB	-22287.64	-0.966	-4625.448	-4626.414		
**THF solvents**							
Step-III B	R- IIIB	-35018.87	-1.518	-5975.445	-5977.263	+1.931	Not Spontaneously occurred
	TS- IIIB	-27558.87	-1.195	-5963.494	-5964.689		
	P- IIIB	-27997.17	-1.214	-5974.118	-5975.332		
Step-IV B	R-IVB	-31459.53	-1.364	-5946.694	-5948.058	+2.543	Not Spontaneously occurred
	TS- IVB	-31464.82	-1.364	-5946.694	-5948.058		
	P- IVB	-709.99	-0.030	-5945.485	-5945.515		
Step-V B	R- VB	-31892.08	-1.382	-5989.070	-5990.452	+1363.687	Not Spontaneously occurred
	TS- VB	-31661.18	-1.372	-5989.070	-5990.442		
	P- VB	-22395.85	-0.971	-4625.794	-4626.765		

### 3.5 Solvent effect on overall reaction progress without catalysis (DABCO) by heat of formation and chemical potential

**Figure 4:**
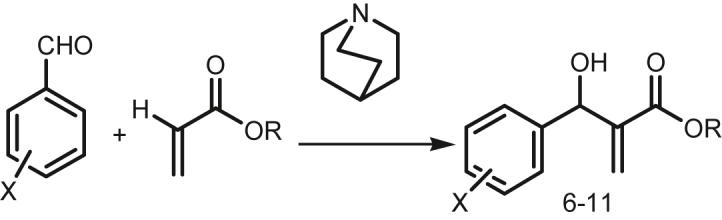
Overall reaction progress pathway.

**Figure 5:**
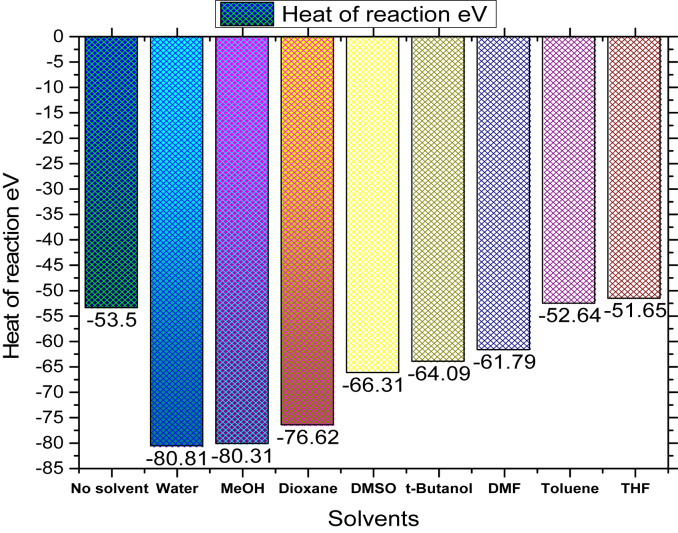
Comparison of heat of reaction in presence of eight solvents.

In Figure 4 represents the overall reaction progress. On this reaction, it has already discussed that DABCO and solvent have a vast convince effects to occur this reaction. In Figure 5 conveys the heat of reaction which is calculated from heat of formation for reactant, and product (attached in Supplementary Table S3). From Figure 5, it is observed that the heat of reaction shows the negative magnitude. First of all, the heat of reaction is accounted for no solvent at -53.5 eV which it is slightly lower than THF and Toluene base solvent but higher for others.

### 3.6 Solvent effect of overall reaction progress without catalysis (DABCO) by Gibbs free energy for reaction

In Table 6, Gibbs free energy of reaction in presence of various solvents has listed in view of reactant and product for reaction Figure 4. The Gibbs free energy is obtained +804.69 eV for no solvent. But variable trends have found when various solvents were applied (attached in supplementary Table S2). The smaller Gibbs free energy for reaction indicates the more reaction occurring possibility. It is surprised that this reaction progress has proceed for solvents of water, DMSO, THF, and toluene as base solvents with no catalyst whereas this reaction is spontaneous process which can be illustrated the DMol^3^ code of material studio through DFT.

**Table 6:** Thermodynamics data in various solvents effect.

Solvents	ΔG for Reactant, eV	ΔG for Product, eV	ΔG for reaction, eV	Reaction possibility
No solvent & DABCO	-25033.38	-24228.69	+804.69	Not Spontaneously occurred
Water	-21409.17	-21622.23	-214.06	Spontaneously occurred
MeOH	-22624.34	-21603.37	+1020.97	Not Spontaneously occurred
Dioxane	-24912.17	-24231.33	+680.83	Not Spontaneously occurred
DMSO	-24683.72	-24905.77	-222.05	Spontaneously occurred
t-Butanol	-24893.33	-24247.92	+645.41	Not Spontaneously occurred
DMF	-24863.58	-24189.01	+673.57	Not Spontaneously occurred
Toluene	-24890.59	-24960.50	-69.91	Spontaneously occurred
THF	-24622.39	-24751.30	-128.91	Spontaneously occurred

**Figure 6:**
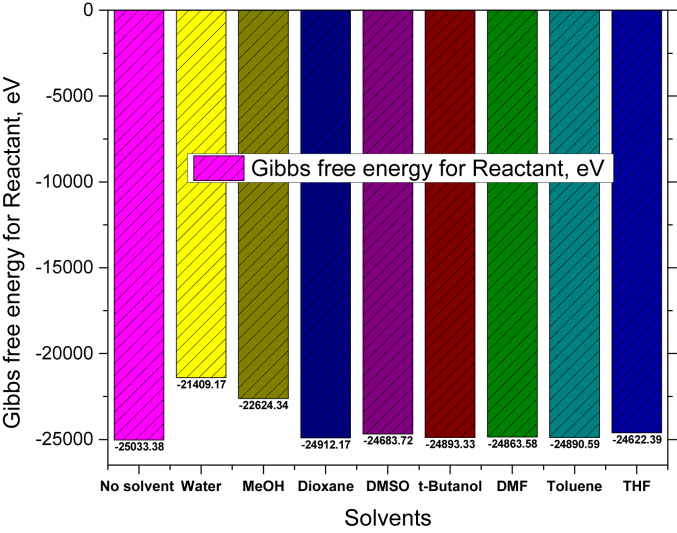
Comparison of Gibbs free energy for reactant in presence of eight solvents.

**Figure 7:**
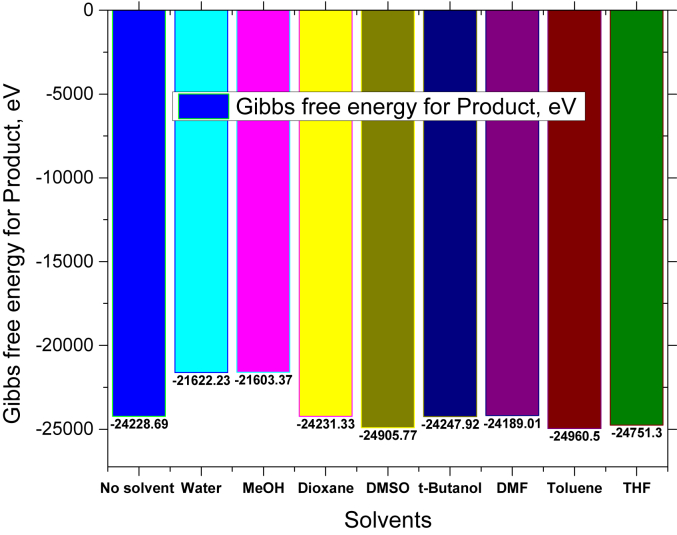
Comparison of Gibbs free energy for reactant in presence of eight solvents.

**Figure 8:**
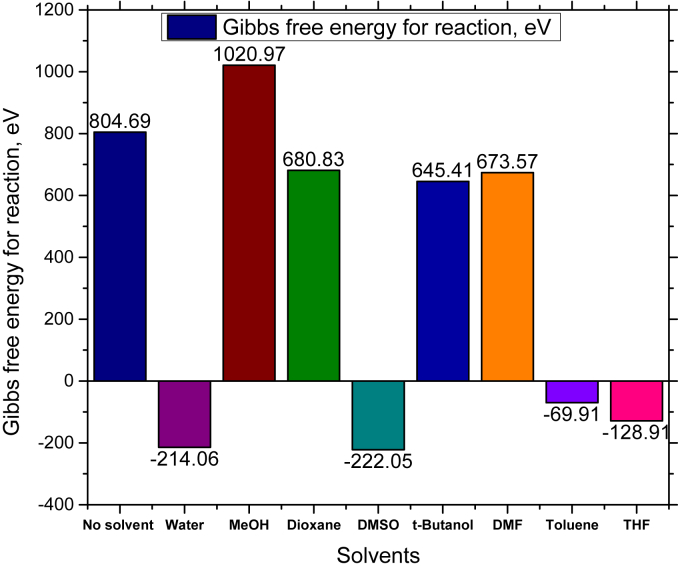
Comparison of Gibbs free energy for reaction in presence of eight solvents.

For making a comparative study among the Gibbs free energy of reactant, product and Gibbs free energy of reaction, the graphical pictures are illustrated in Figures 6, 7, 8. First of all, the Gibbs free energy has abated for reactant in using solvent instead of from no solvent where it is smaller for water and MeOH shown in Figure 6. Next, this is similar phenomena for product shown in Figure 7. However, Figure 8 illustrates the Gibbs free energy for reaction while four solvents, for example water, DMSO, toluene and THF play the vital role for progressing the reaction.

### 3.7 Stereochemistry of product

**Figure 9:**
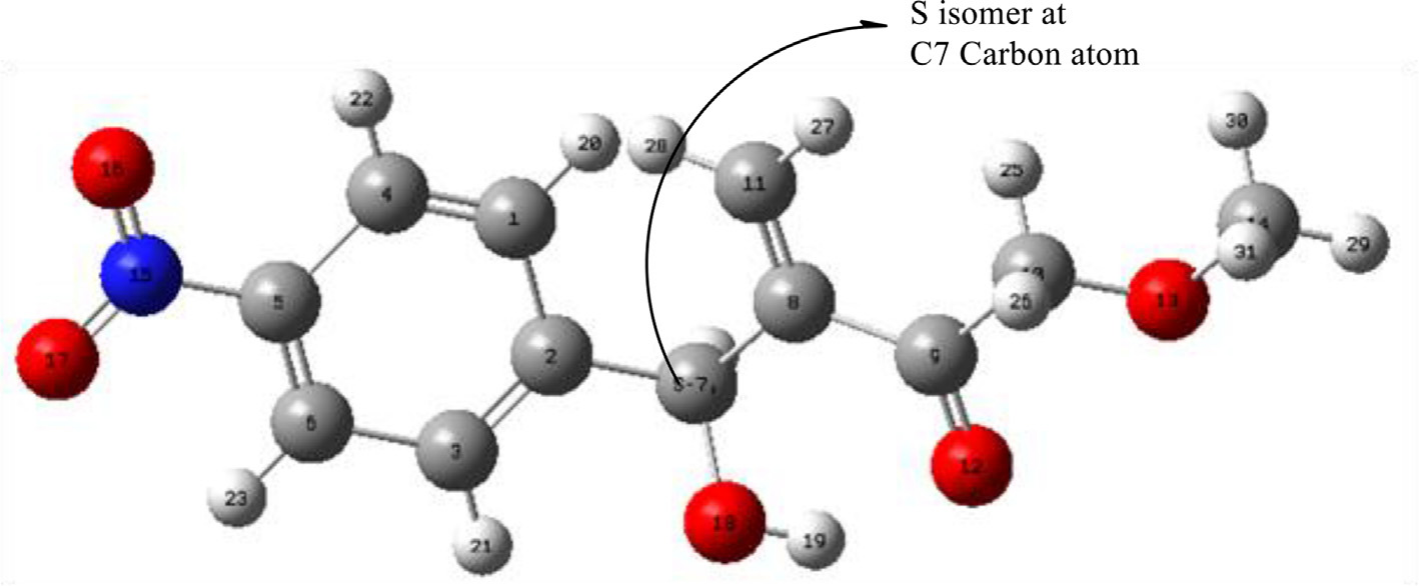
Sterio-centre for product 7.

For developing the stereochemistry of synthesized product, the Gaussian 16 software was used. At first, the product was optimized through DFT functionals for molecular geometry and optimization. After optimization, it was calculated the stereo isomer for products, shown in Figure 9 and Figure S1 (supplementary file). All of the products are *S* isomer in C-7 carbon atom which is also a chiral carbon of alkyl chain.

### 3.8 Chemical descriptors and HOMO and LUMO

Chemical parameters have served as a major catalyst for determining the chemical stability of any compound and their structural geometry. Table 7 describes the different values of these parameters, such as ԑLUMO, HOMO, ionization potential, electron affinity, ԑHOMO ԑLUMO gap, chemical potential, hardness, electrons activity, electrophilicity, and softness. Table 7 shows that the value of LUMO is less than three times that magnitude of HOMO. Thus, the electrophilic groups of all these compounds tend to be much more attracted in the position of higher electronic denser portion. All these, energy gaps as expressed the ϵHOMO ϵLUMO gap between two orbitals are seen to be around 7.0 eV or slightly more. As aliphatic chain is occurred in their body, are caused for some variations in their chemical activity. The first is that the chemical potential energy levels of the chemical potential are close to -6.00 eV. There is a great deal of similarity between the compound and the chemical descriptors due a vast changing in their body chain. Their energy levels i.e. hardness values are around 3.0 eV while softness values are around 0.28 eV which indicates maximum chemical activity for organic compounds [39, 43, 50, 51, 52, 53].

**Table 7:** Frontier molecular orbitals and Reactivity descriptor analysis.

	ϵLUMO, eV	ϵHOMO, eV	Ionization potential (I), eV	Electron affinity (A), eV	ϵHOMO ϵLUMO gap, eV	Chemical potential (μ), eV	Hardness (η), eV	Electrons activity (χ), eV	Electrophilicity (ω), eV	Softness (S), eV
**06**	-9.78	-2.3	9.780	2.300	7.480	-6.040	3.740	6.040	4.877	0.267
**07**	-9.87	-2.46	9.870	2.460	7.410	-6.165	3.705	6.165	5.129	0.270
**08**	-10.1	-2.41	10.100	2.410	7.690	-6.255	3.845	6.255	5.088	0.260
**09**	-9.92	-2.48	9.920	2.480	7.440	-6.200	3.720	6.200	5.167	0.269
**10**	-9.77	-2.68	9.770	2.680	7.090	-6.225	3.545	6.225	5.466	0.282
**11**	-9.8	-2.47	9.800	2.470	7.330	-6.135	3.665	6.135	5.135	0.273

### 3.9 Frontier molecular orbital diagram in term of HOMO and LUMO

**Figure 10:**
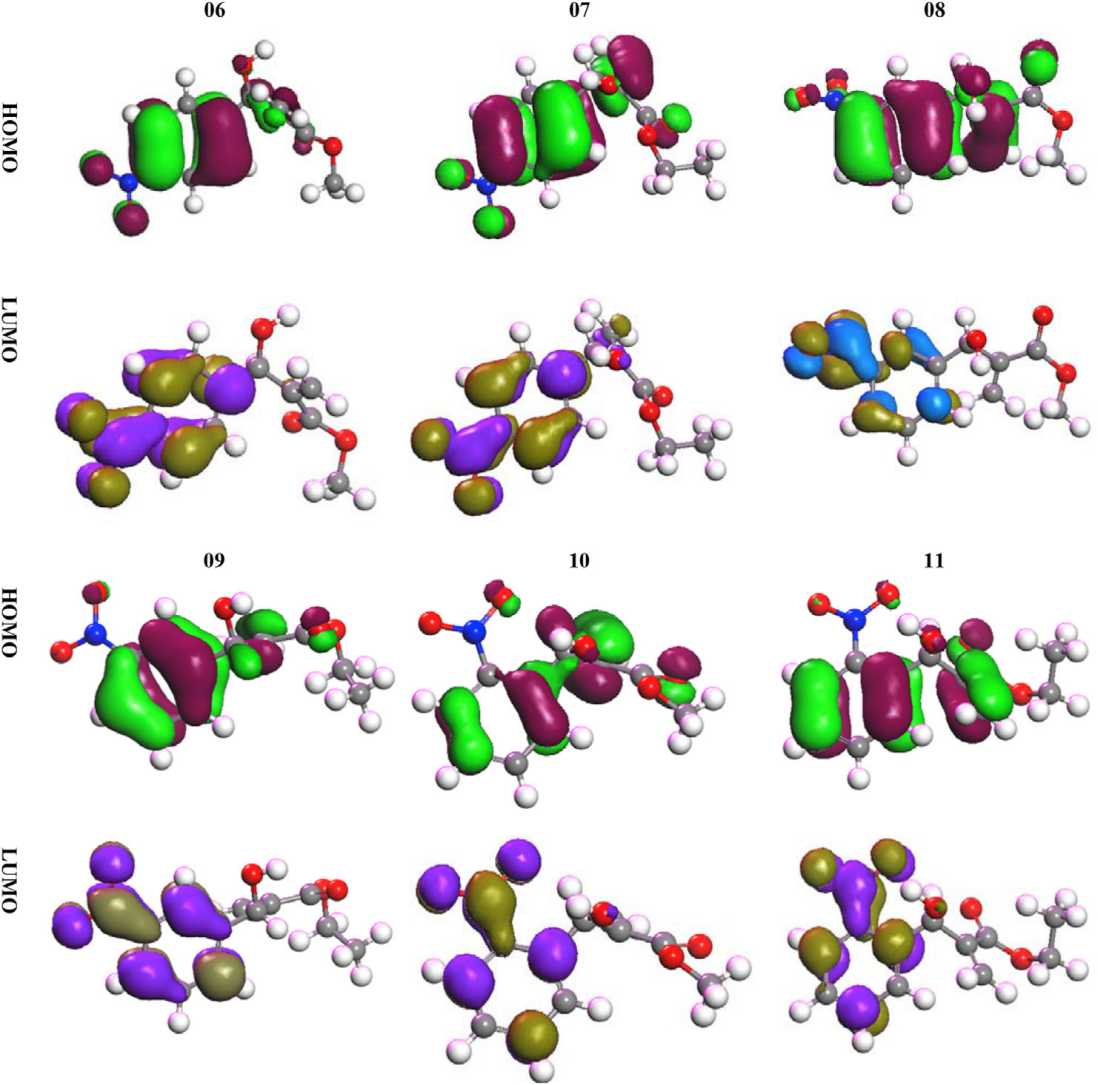
HOMO, LUMO orbital diagram for **(6–11)**.

The LUMO and HOMO orbital diagrams indicate the most imperative parts of a molecule that can be joined by electrophilic and nucleophilic groups, resulting in a variety of chemical reactions. The LUMO and HOMO orbital diagrams are illustrated in Figure 10. First of all, the color green refers to the positive part of the orbital and the maroon color refers to the negative part. In the case of LUMO, it has been shown that lemon refers to the positive part and purple refers to the negative part. As it can be seen from Figure 10, these additions usually have three parts, benzene cycle, alkyl group, nitro group at side chain and ester group with negative atoms. Now, Figure 10 is how the reactivity of these functional groups but can be easily determined through this orbital picture. First, in the case of HOMO, alkyl chains and benzene rings are found more or less in this part of the world, even with the ester group, but the LUMO part is only found in the nitro group benzene ring and its surrounding. Thus, it is clear that the functional group of the compound has a far-reaching effect for the addition of nucleophilic groups, and the nitro group for the addition of electrophilic groups.

### 3.10 Electrostatic potential charge distribution

**Figure 11:**
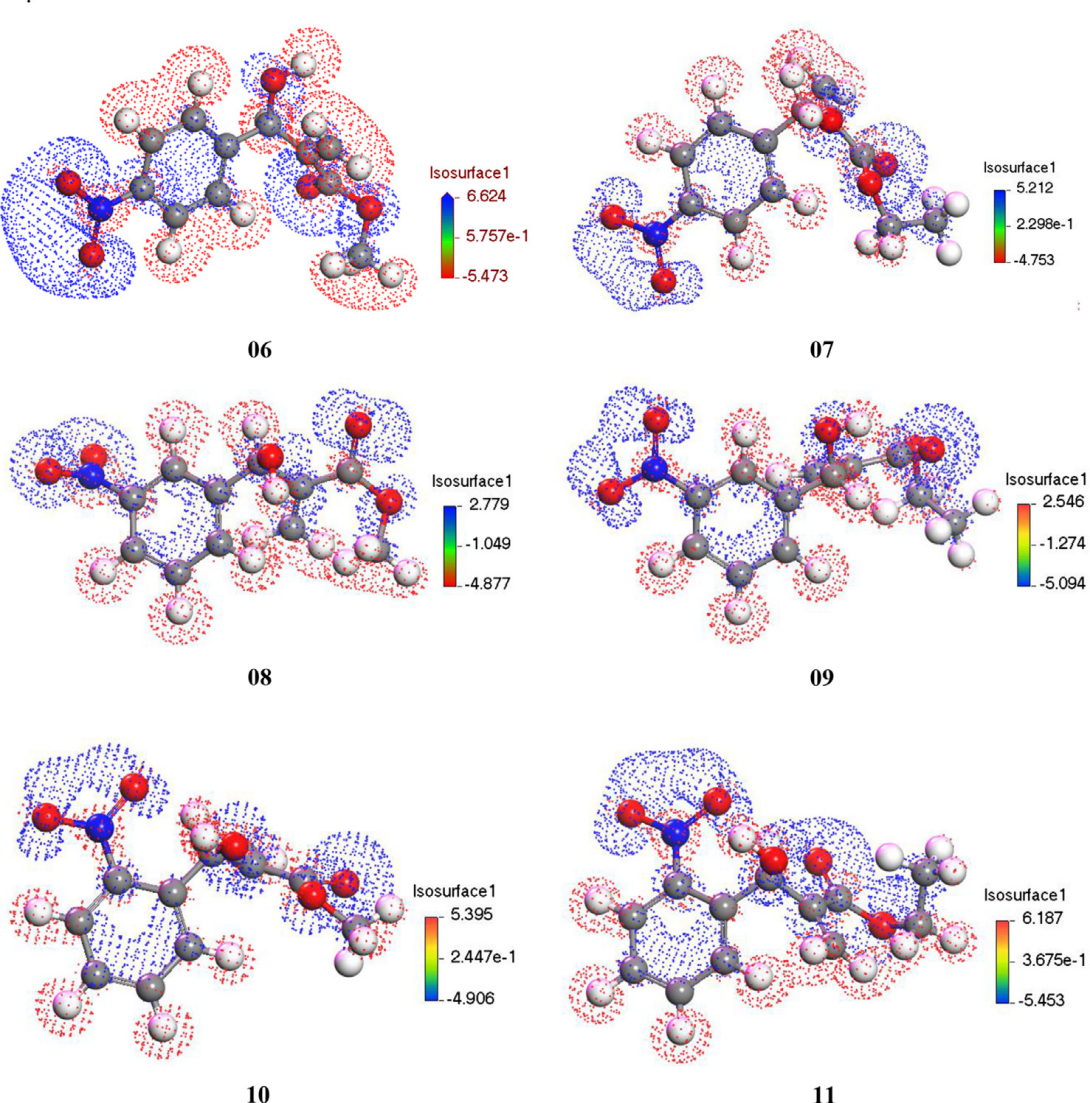
Electrostatic Potential charge distribution map.

The following electrostatic potential map shows how the molecules are distributed inside the charges through its full body. It may be influenced to form the weak bonds that are formed for the substance to bind to proteins as attracting to the hydrophobic and hydrophilic charges for which the charges are produced here for responsible that fact. In this context, by properties, it can be revealed whether matter has biological activity. The image below Figure 11 shows that there is a large gap between the positive and negative charges inside the substance as shown on the scale. This means that as the gap widens, there is a greater tendency for charges to be distributed and involved.

## 4 Conclusion

The synthesis of alkyl 2-[hydroxyl (nitrophenyl) methyl] acrylate in one step under mild conditions has been discussed in details of catalytic nature at optimized temperature and suitable solvents. To kick off, THF solvent is obtained the best solvent among 1, 4-dioxane, tetrahydrofuran (THF), dimethylsulfoxide (DMSO), dimethylformide (DMF), dichloromethane (DCM), toluene, acetonitrile, *t*-butanol and methanol, Dioxane, DCM, *t*-butanol. Next, 20 mol% of DABCO could be sustainable catalyst among 10%, and 100 % in presence of THF solvent. Moreover, this reaction does no precede in presence of Et_3_N or C_2_H_5_ONa or C_4_N_9_OK or (CH_3_)_3_COK or Pyridine catalysts having the THF solvent. For occurring this reaction in alkyl 2-[hydroxyl (nitrophenyl) methyl] acrylate and its derivates (**06–11)**, it might be revealed that 4-position of nitro group in aryl acrylate is highly reactive for reactant due to low static hindered force occurring the proton transferring. Then, the synthesized acrylate derivates (**06–11)** were characterized by 1H NMR, 13C NMR FTIR and UV data. Finally, with the computational evidence, it could be said that this reaction follows the 2^nd^ order reaction mechanism with THF solvent in presence of DABCO catalyst at room temperature for formation of carbon-carbon single bond between aryl acrylates and aldehydes. The control of stereochemistry of BH adducts and it might develop chemical transformation of –OH group at chiral centre and addition reaction at double bond. By the computational calculation, it was found that the synthesized acrylate derivates (**06–11)** are the *S*-stereoisomer at C7 carbon atom. At last, the HOMO and LUMO and energy gaps make the further study for the biological significances as bioactive molecules.

## Declarations

### Author contribution statement

Laila Arifun Nahar: Conceived and designed the experiments; Performed the experiments.

Ajoy Kumer: Performed the experiments; Analyzed and interpreted the data; Wrote the paper.

Md Wahab Khan: Contributed reagents, materials, analysis tools or data.

### Funding statement

This work was supported by 10.13039/501100009500Bangladesh University of Engineering & Technology (BUET), Dhaka-1000, Bangladesh.

### Data availability statement

Data included in article/supplementary material/referenced in article.

### Declaration of interests statement

The authors declare no conflict of interest.

### Additional information

No additional information is available for this paper.

## Appendix A Supplementary data

Modififed_Supplementary file_2_10_21 Modififed_Supplementary file_2_10_21
