# Supplementary material for: Investigation of catalytic effect on carbon-carbon bond formation by Baylis-Hillman (BH) reaction between (2/3/4)-nitro-arylaldehyde and alkylacrylates and computational approaches through DFT functional
Source: Heliyon. 2021 Oct 15;7(10):e08139. doi: 10.1016/j.heliyon.2021.e08139 (PMC8529513; doi:10.1016/j.heliyon.2021.e08139)
Supplement: Modififed_Supplementary file_2_10_21 [file mmc1.docx]

| Table S1: Reactants, products and yields | | | | |
| --- | --- | --- | --- | --- |
| Entry | **Aldehyde** | **Acrylates** | **Product** | **^a^Yield%** |
| 1. |   **1** |   **4** |   **6** | 90 |
| 2. |   **1** |   **5** |   **7** | 90 |
| 3. |   2 |   4 |   8 | 83 |
| 4. |   2 |   5 |   9 | 70 |
| 5. |   3 |   4 |   10 | 80 |
| 6. |   3 |   5 |   11 | 84 |

| **S.L** | **Synthesized molecules** | **Result** |
| --- | --- | --- |
| **6** | 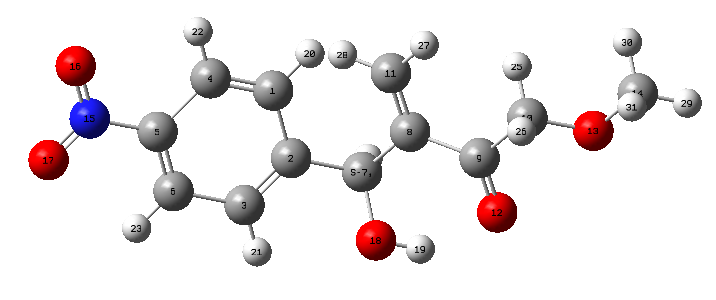 | **S** isomer  C7 Carbon atom |
| **7** | 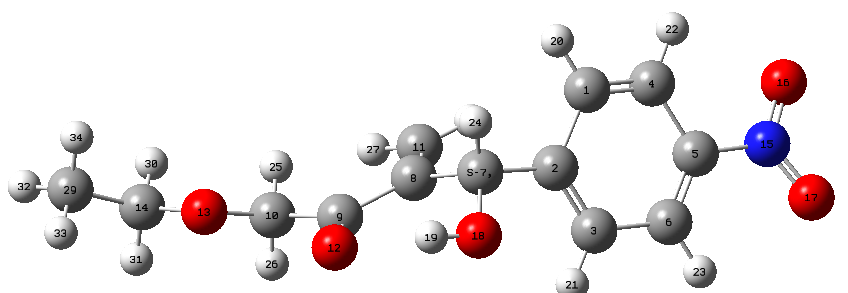 | **S** isomer  C7 Carbon atom |
| **8** | 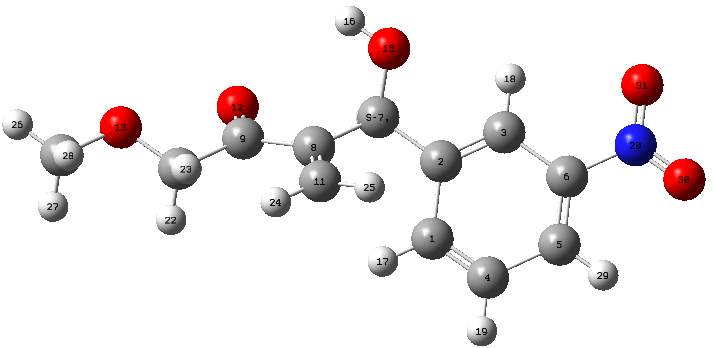 | **S** isomer  C7 Carbon atom |
| **9** | 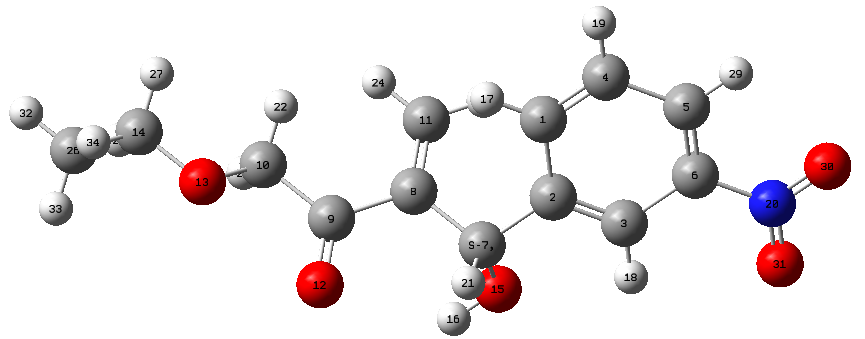 | **S** isomer  C7 Carbon atom |
| **10** | 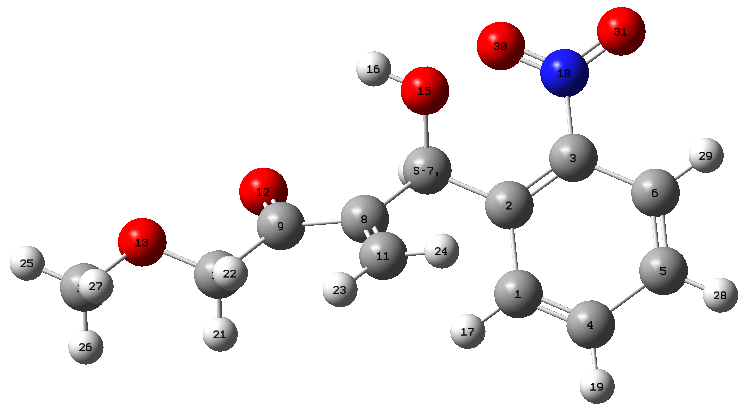 | **S** isomer  C7 Carbon atom |
| **11** | 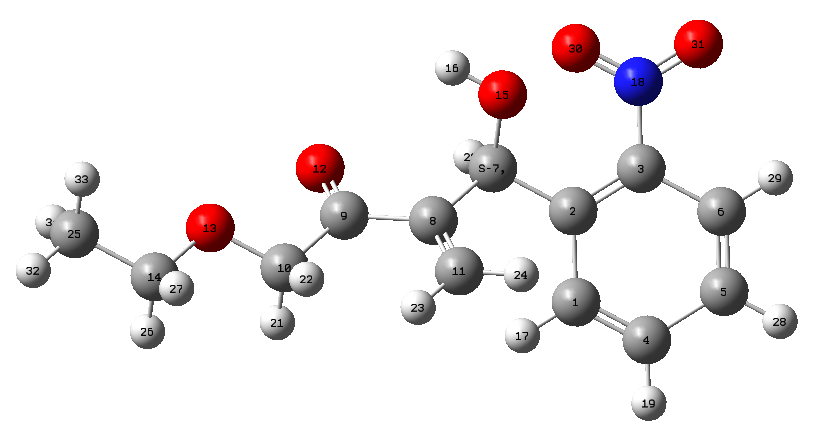 | **S** isomer  C7 Carbon atom |

**Figure: S1: stereo centre of optimized structure**

**Compound-06**


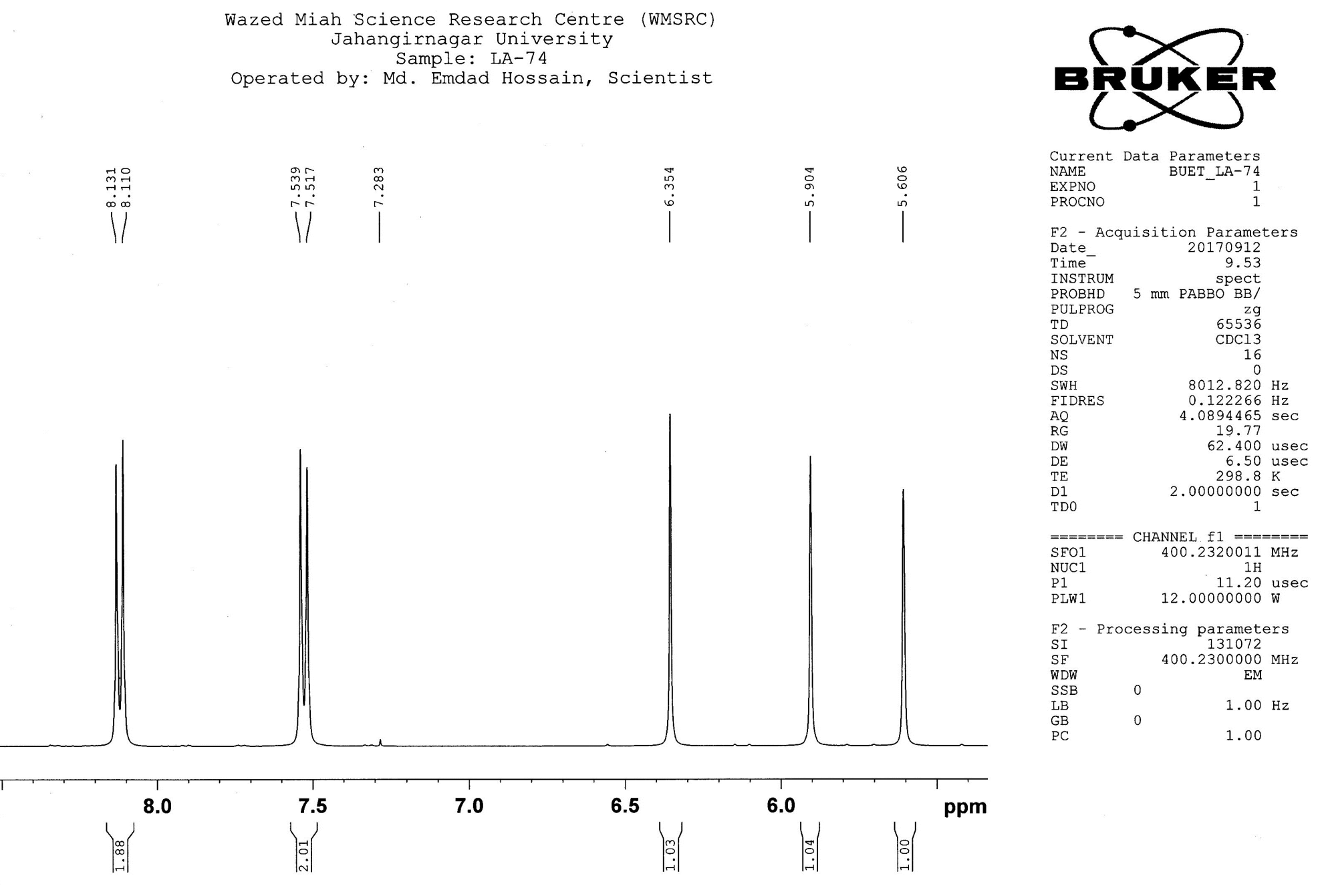

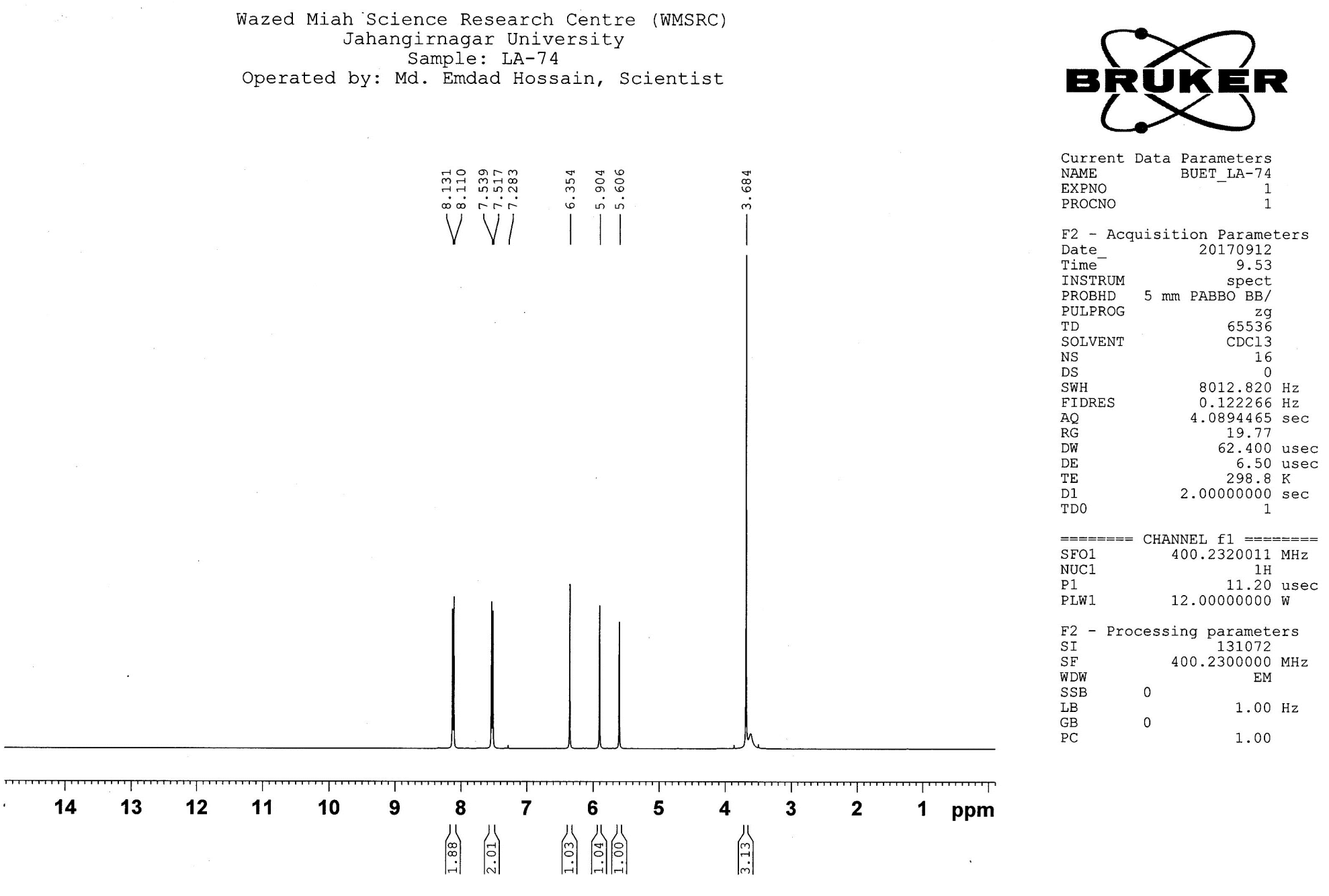

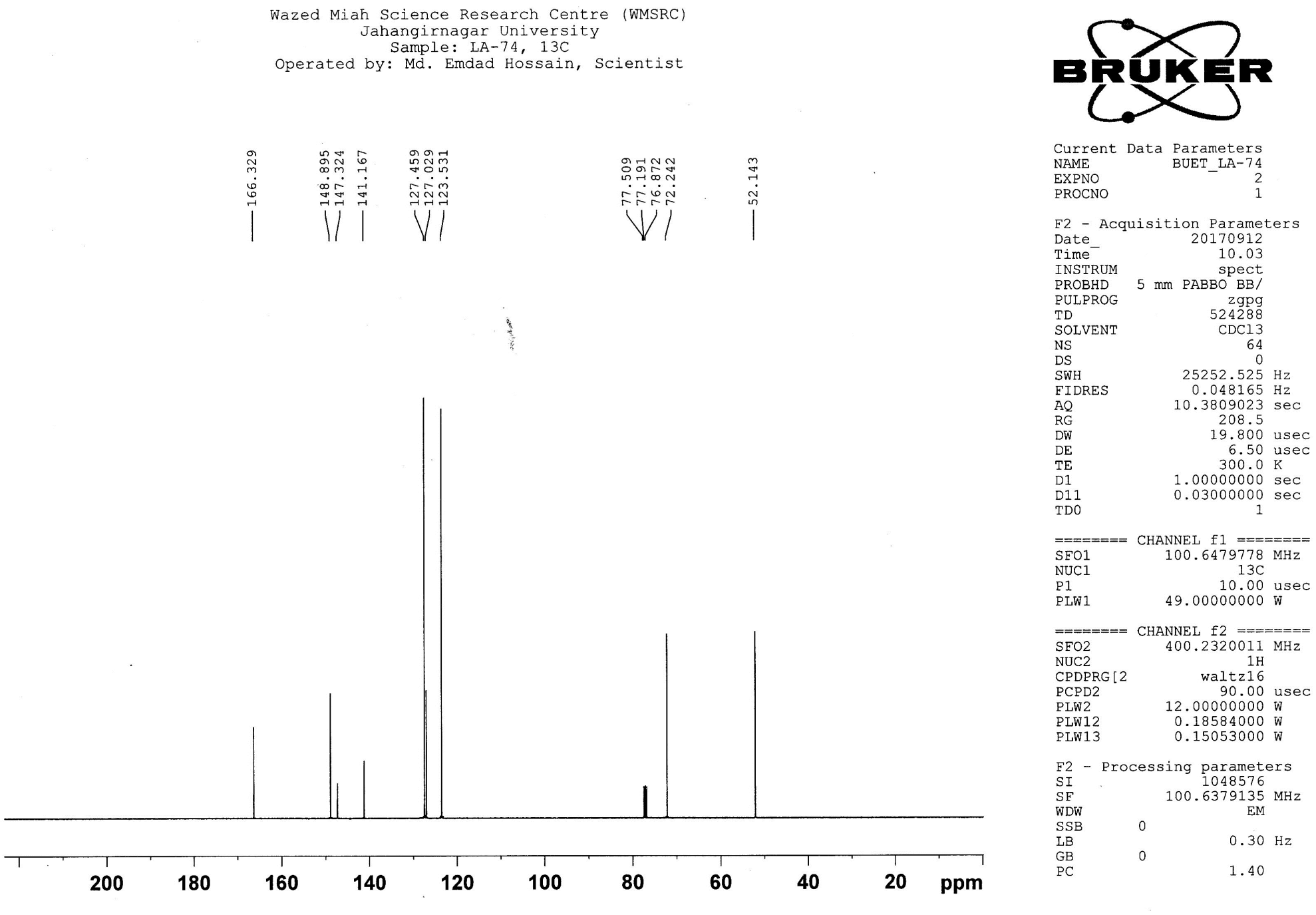

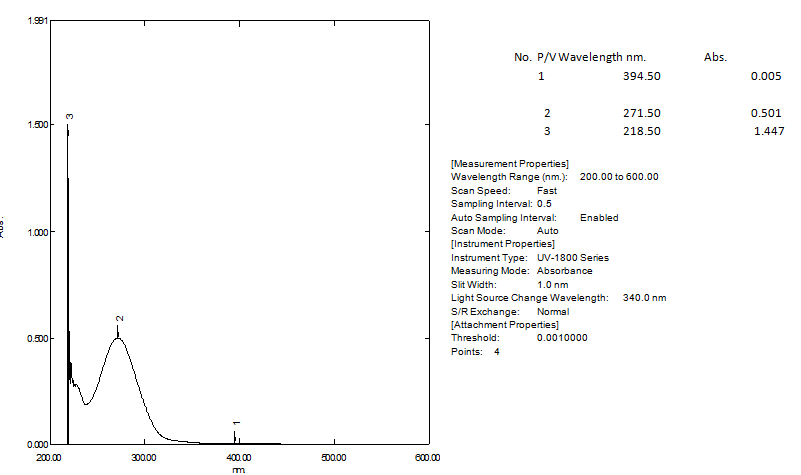


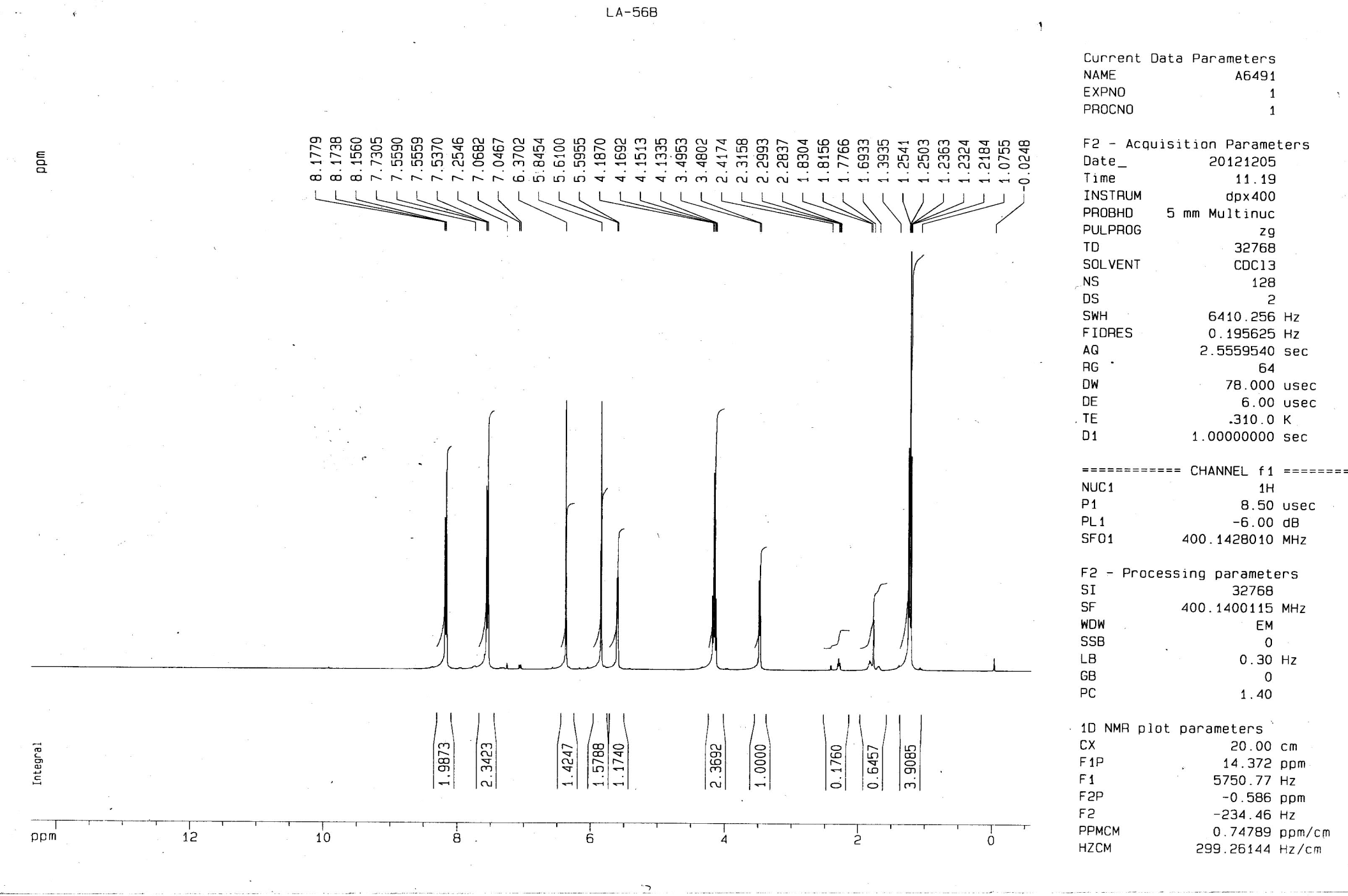
**Compound-07**

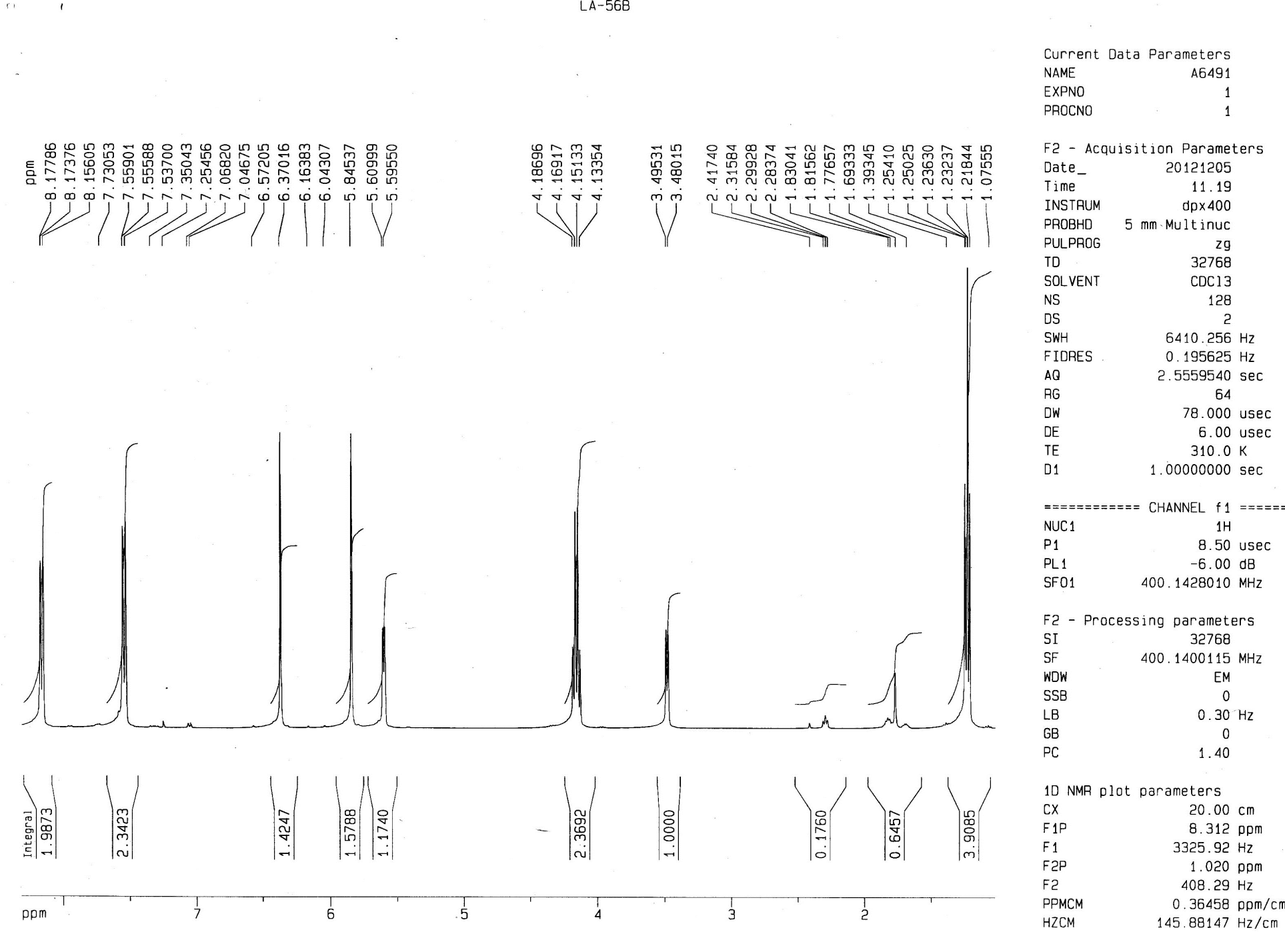

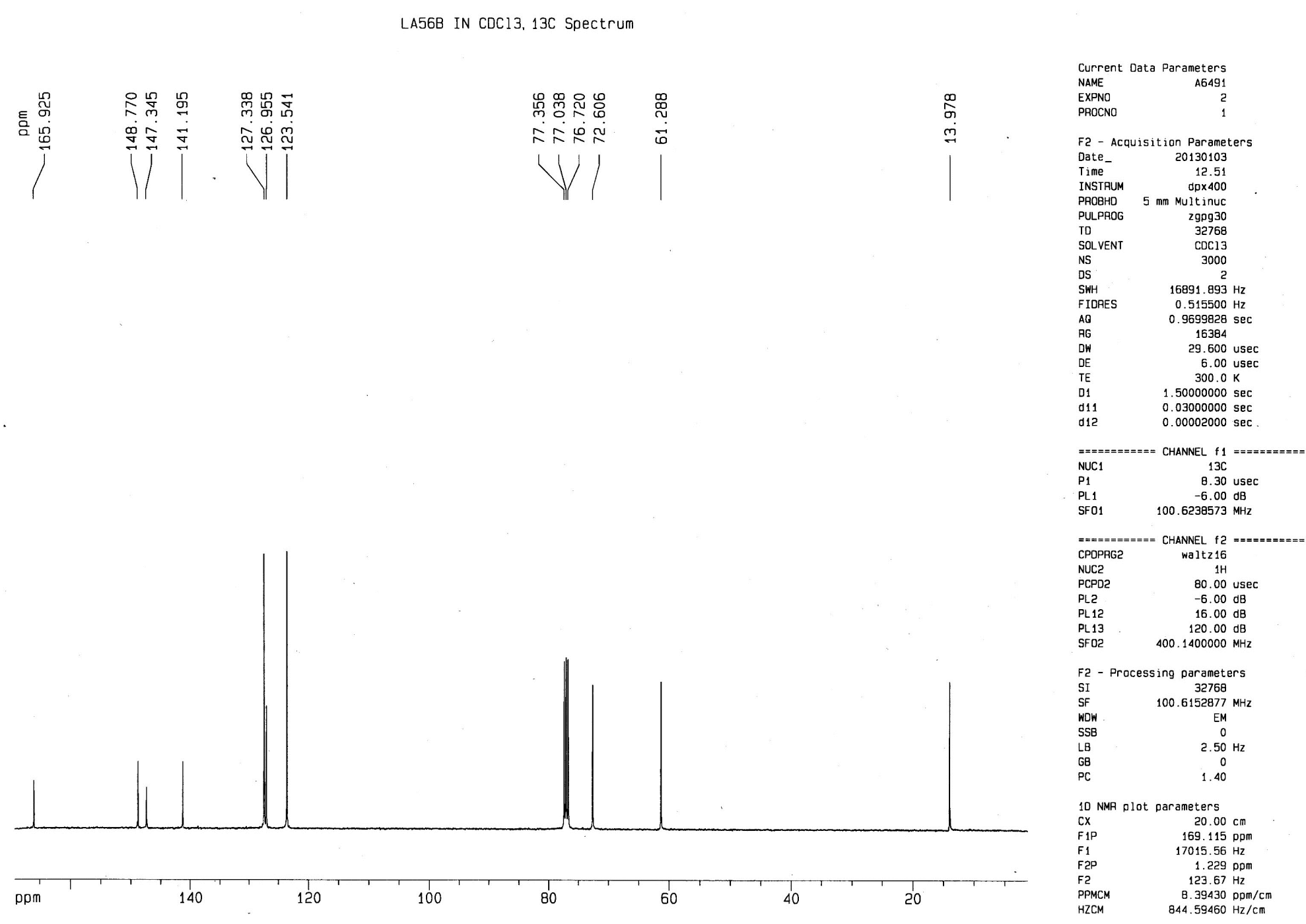

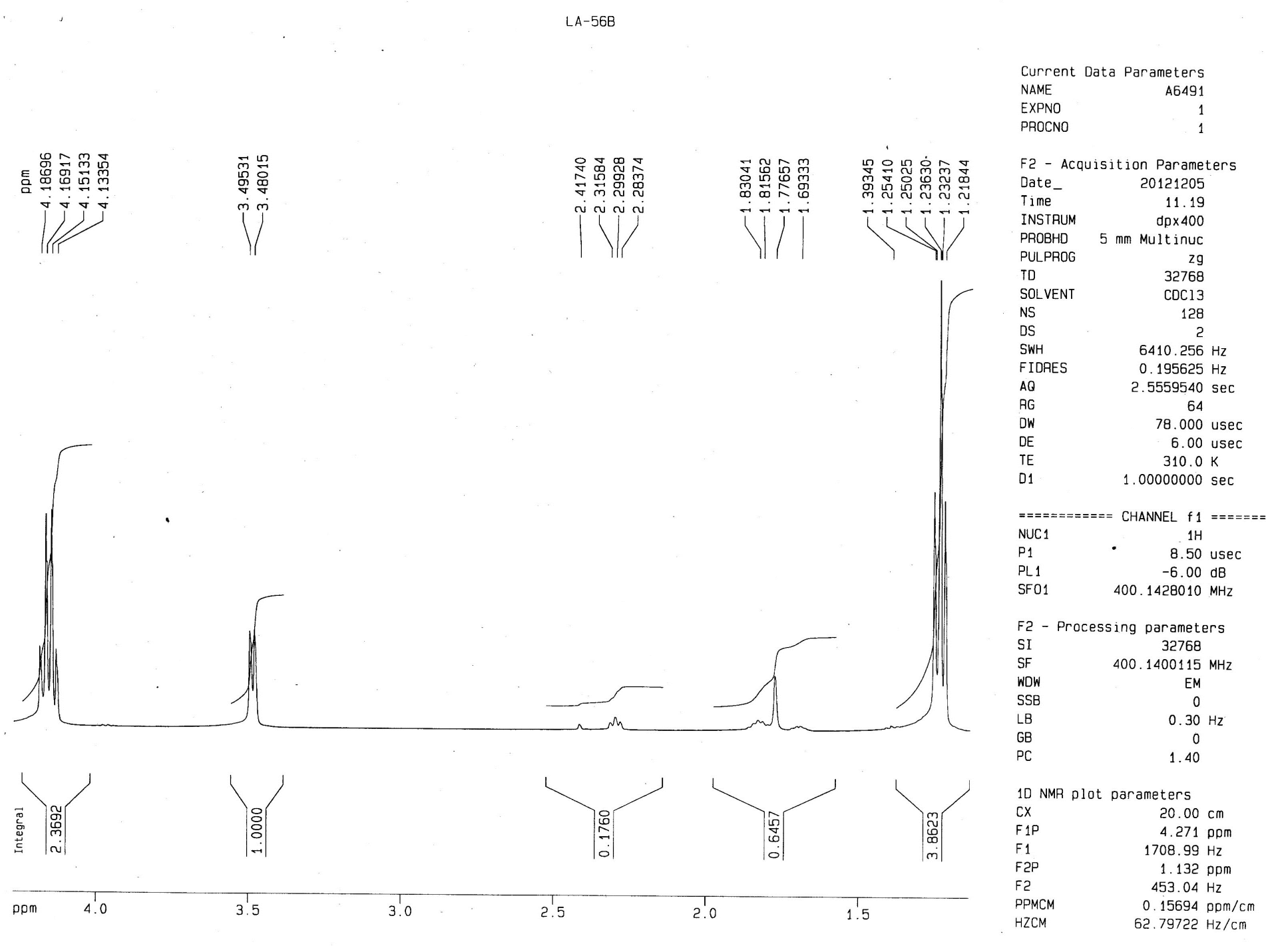

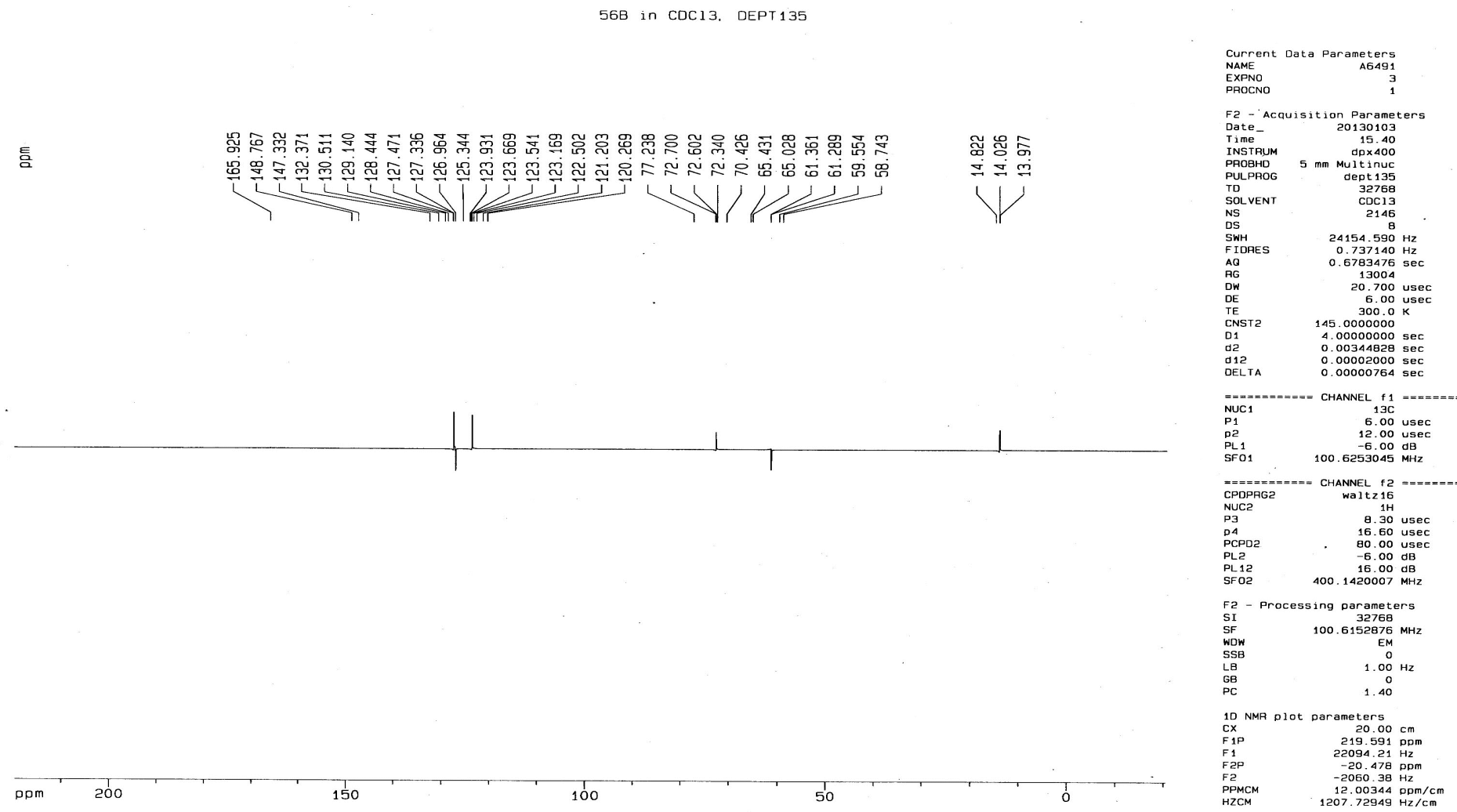

 No. P/V Wavelength nm. Abs.

1 336.00 0.027

2 273.00 0.901

3 218.80 3.035

4 214.40 0.364

5 204.60 -1.818

[Measurement Properties]

Wavelength Range (nm.): 200.00 to 500.00

Scan Speed: Fast

Sampling Interval: 0.2

Auto Sampling Interval: Enabled

Scan Mode: Auto

[Instrument Properties]

Instrument Type: UV-1800 Series

Measuring Mode: Absorbance

Slit Width: 1.0 nm

Light Source Change Wavelength: 340.0 nm

S/R Exchange: Normal

[Attachment Properties]

Attachment: None

[Operation]

Threshold: 0.0010000

Points: 4

**Compound-08**
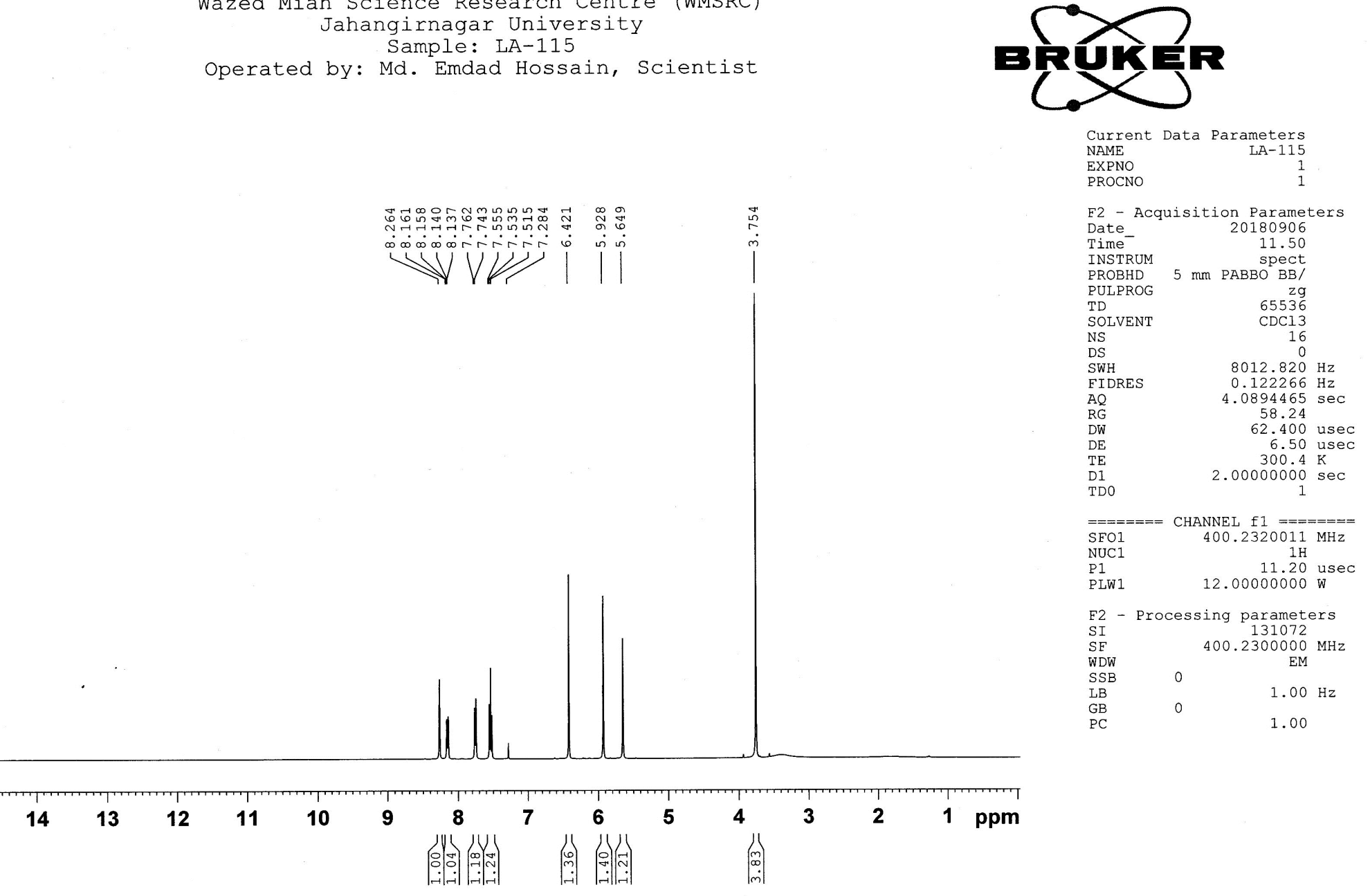

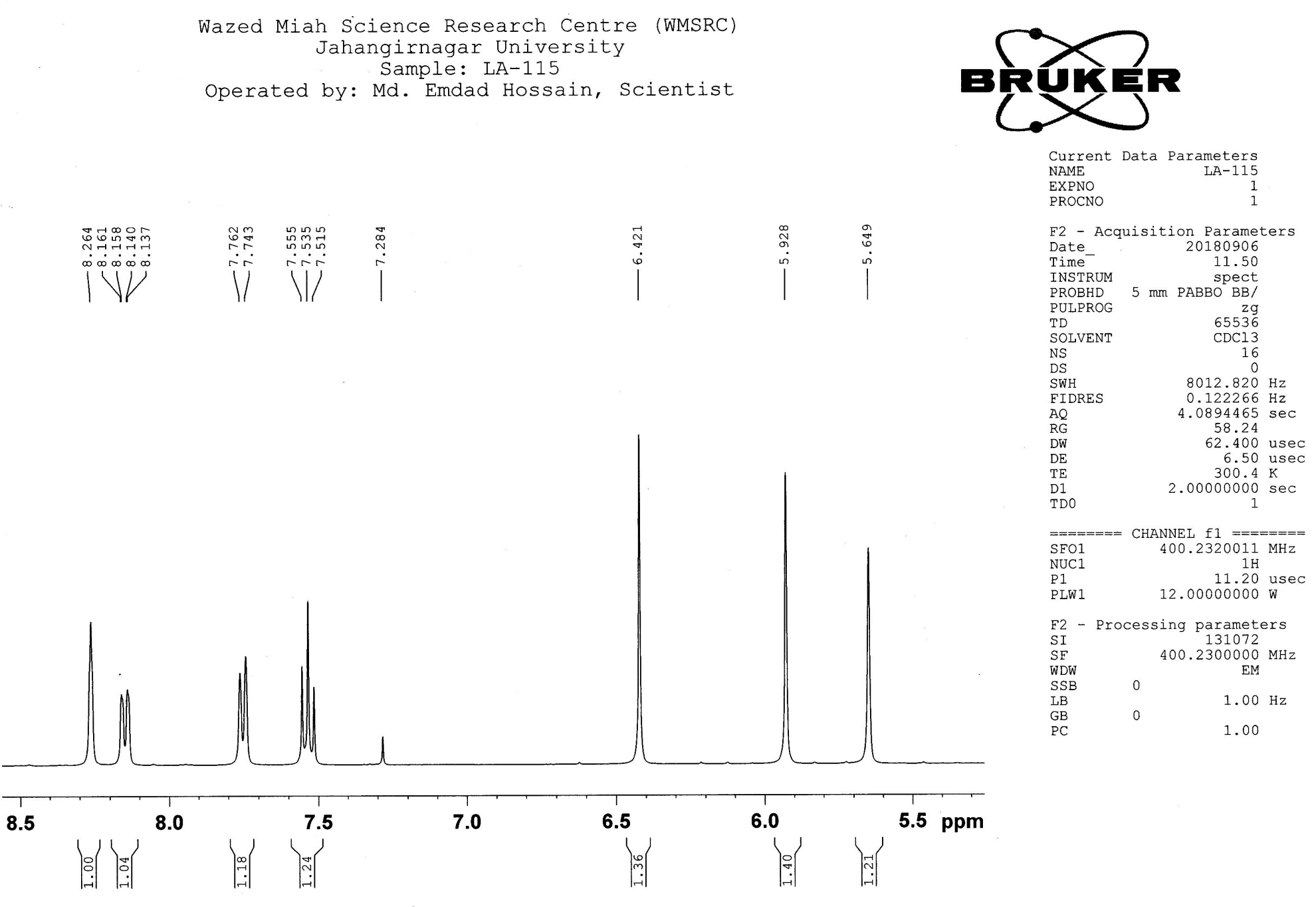

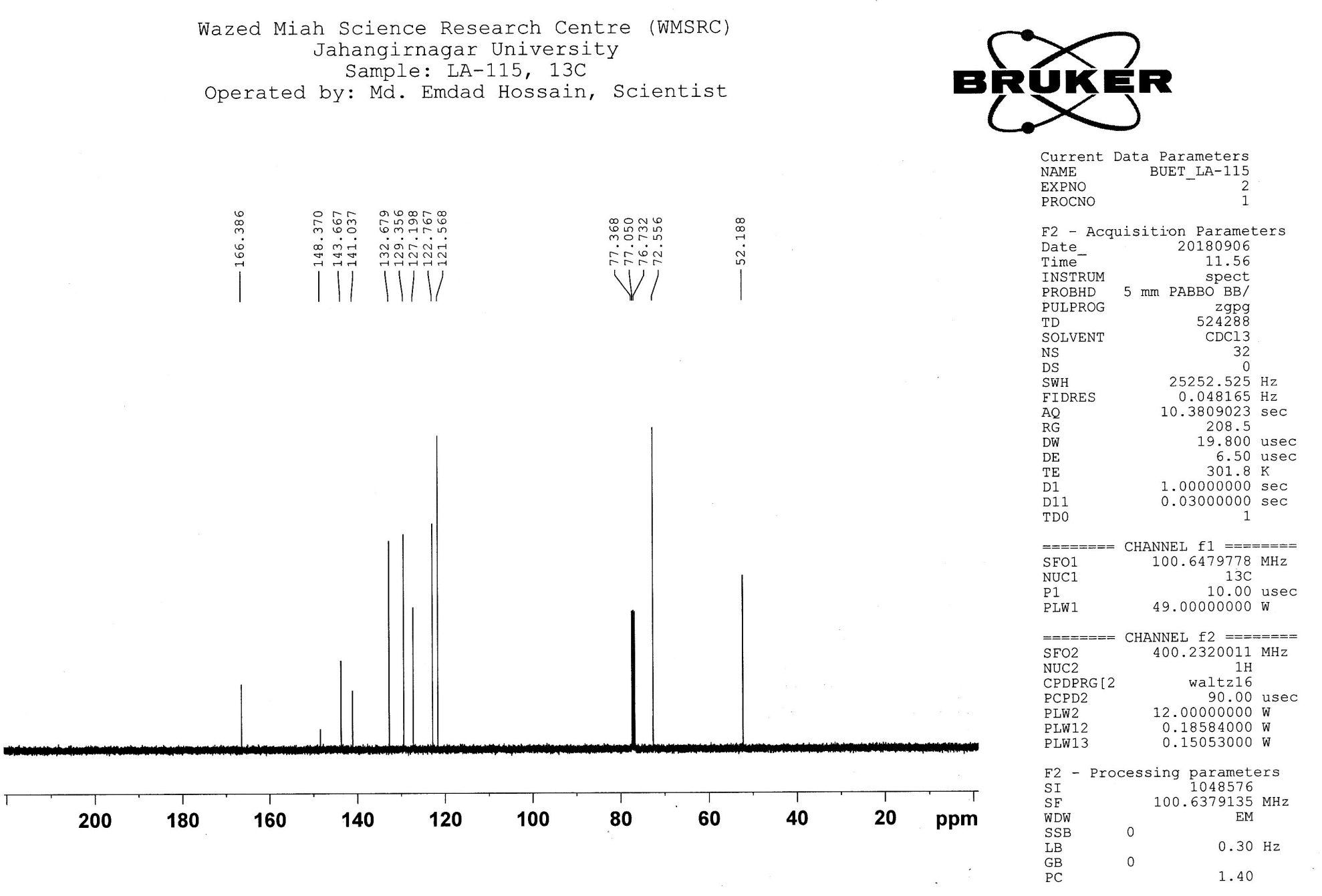

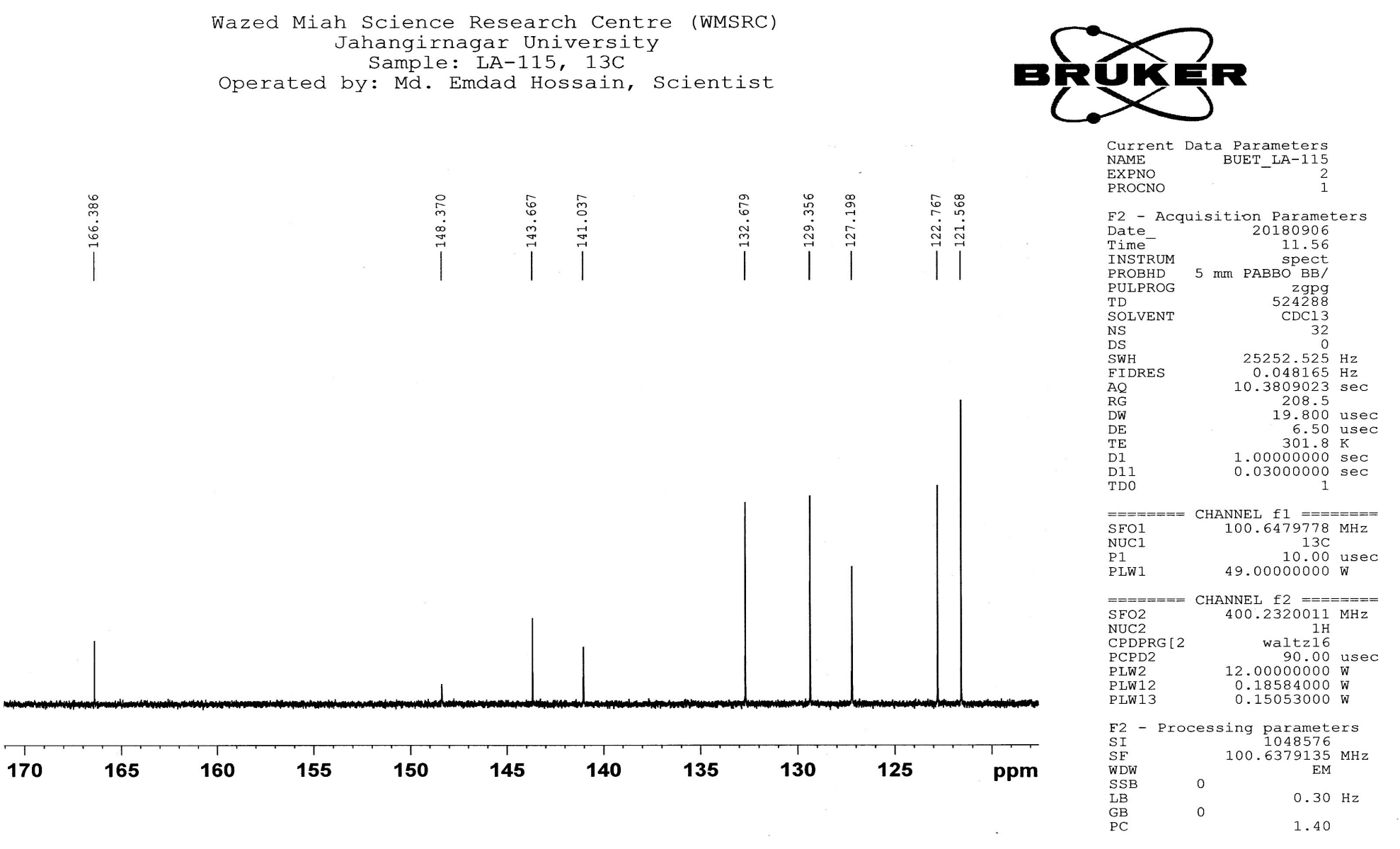


No. P/V Wavelength nm. Abs.


1 676.50 0.013

2 263.50 0.605

3 224.50 1.543

[Measurement Properties]

Wavelength Range (nm.): 200.00 to 800.00

Scan Speed: Fast

Sampling Interval: 0.5

Auto Sampling Interval: Enabled

Scan Mode: Single

[Instrument Properties]

Instrument Type: UV-1800 Series

Measuring Mode: Absorbance

Slit Width: 1.0 nm

Light Source Change Wavelength: 340.0 nm

S/R Exchange: Normal

[Attachment Properties]

Attachment: None

[Operation]

Threshold: 0.0010000

Points: 4

**Compound-0**
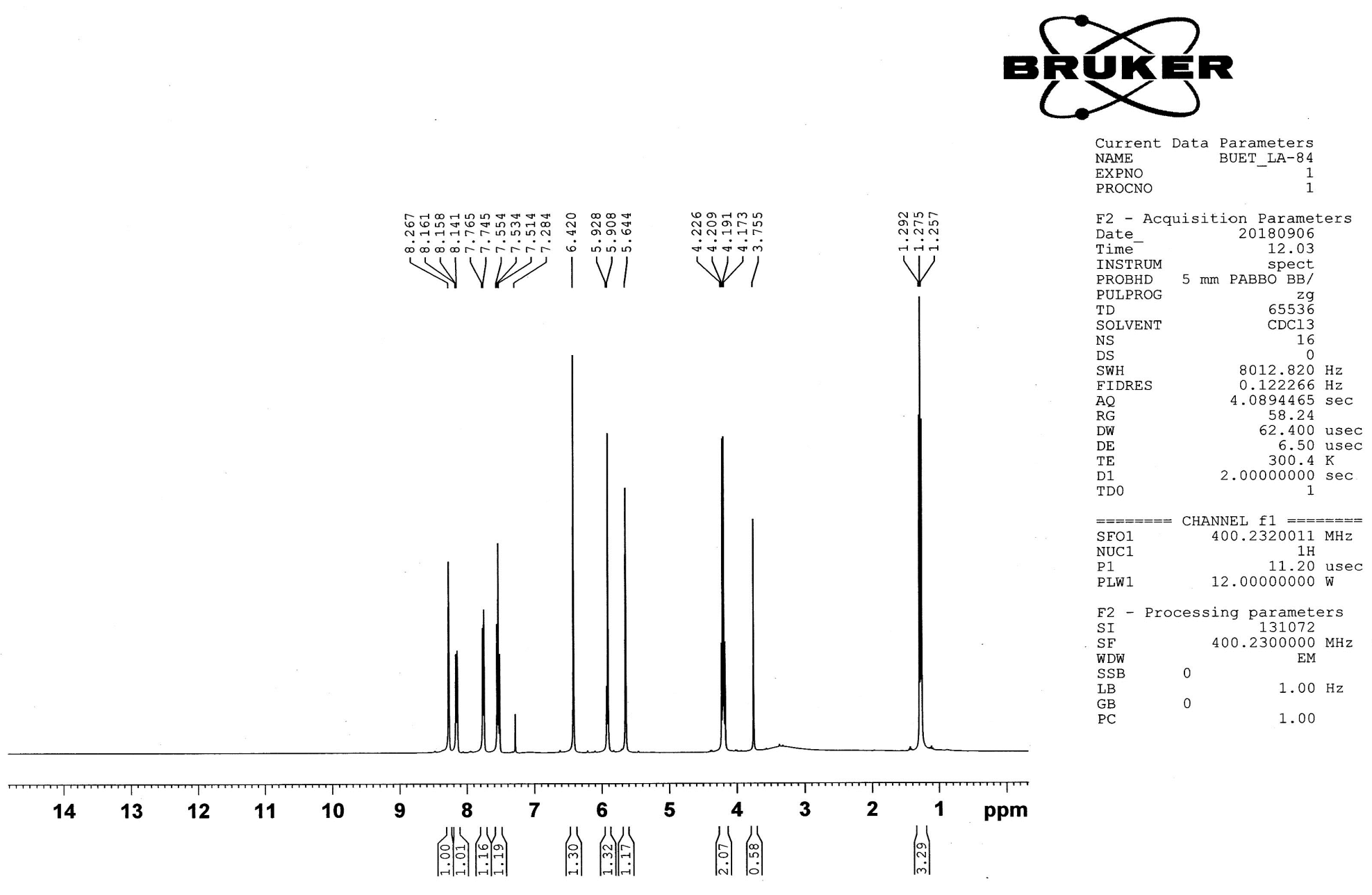

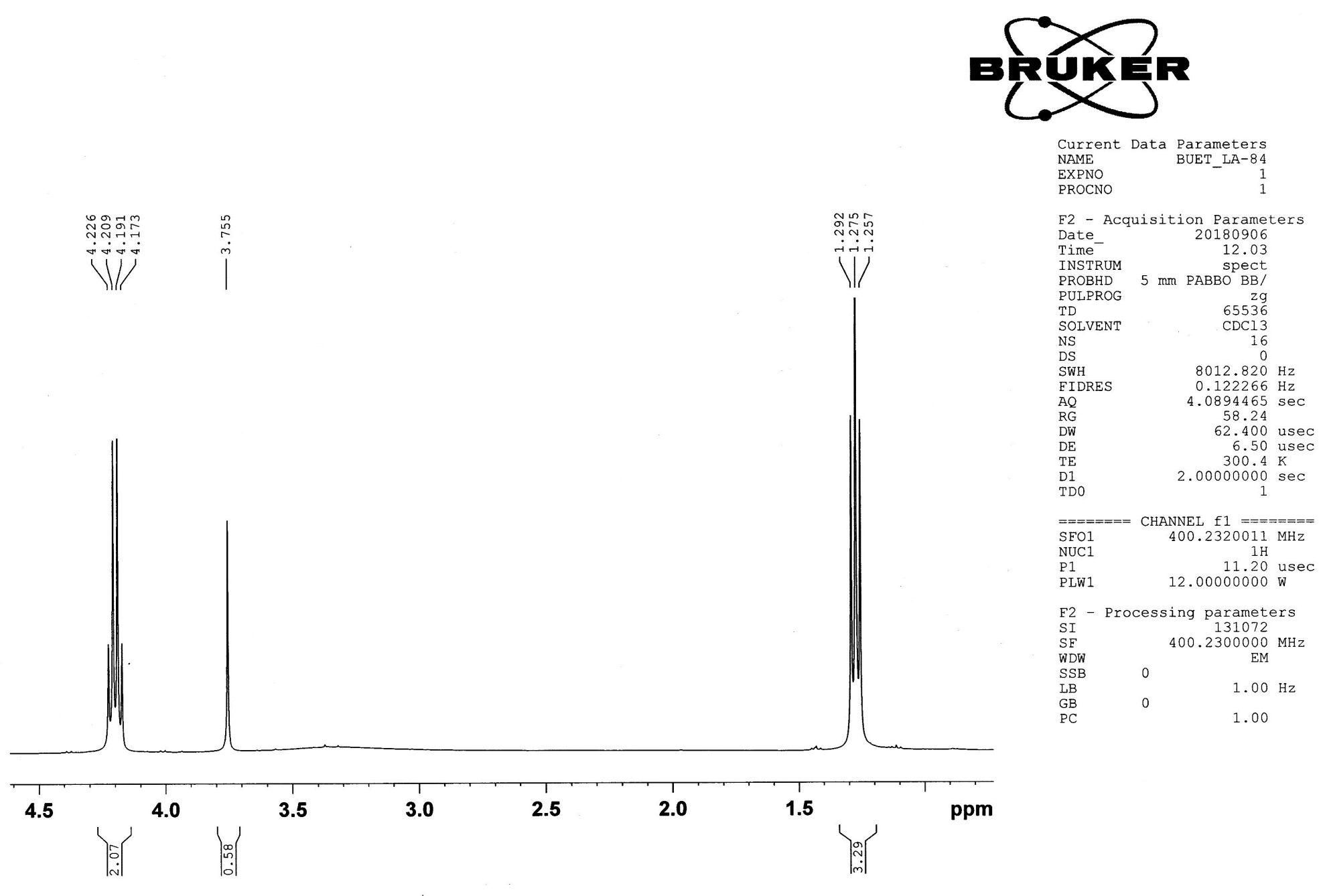
**9**

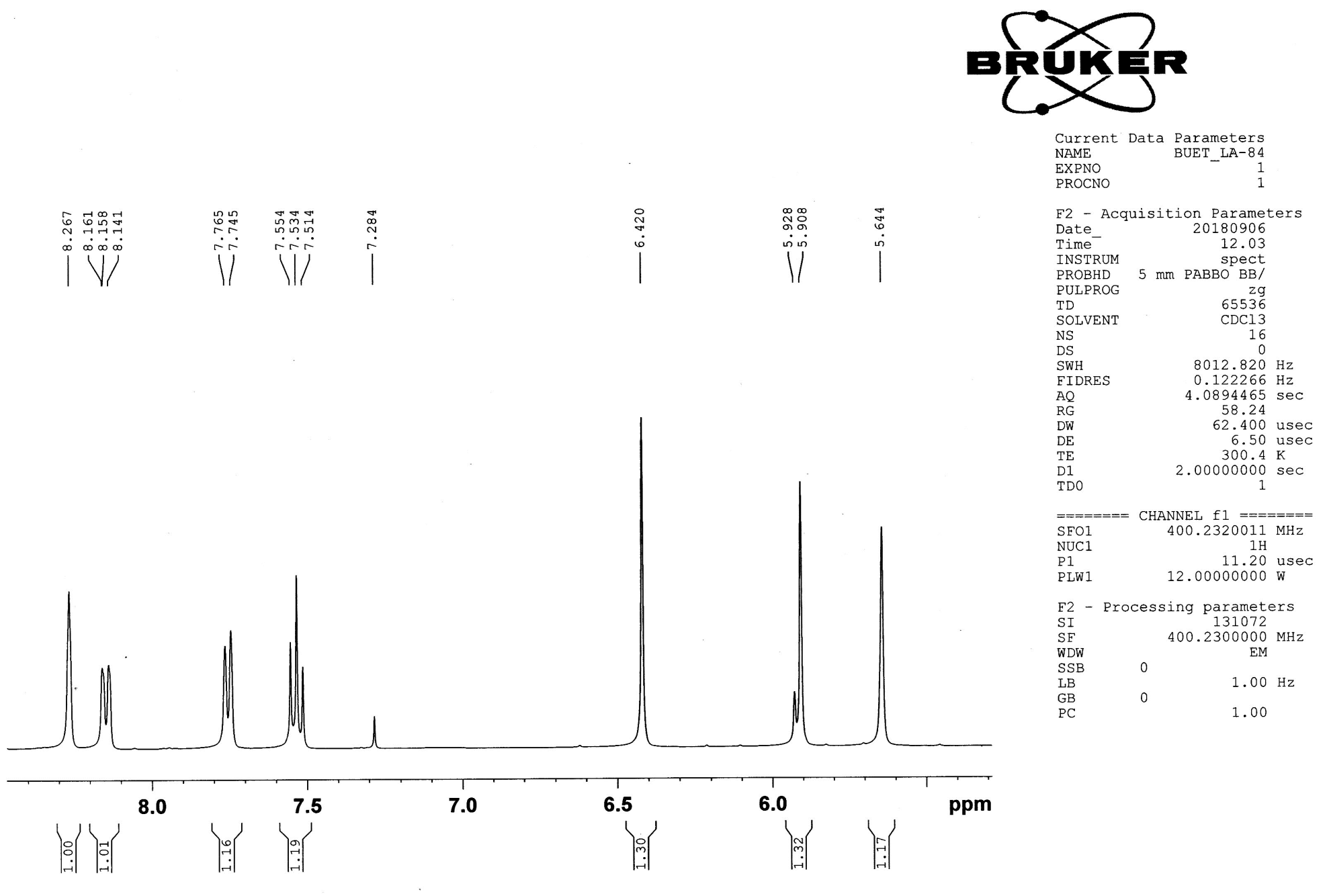

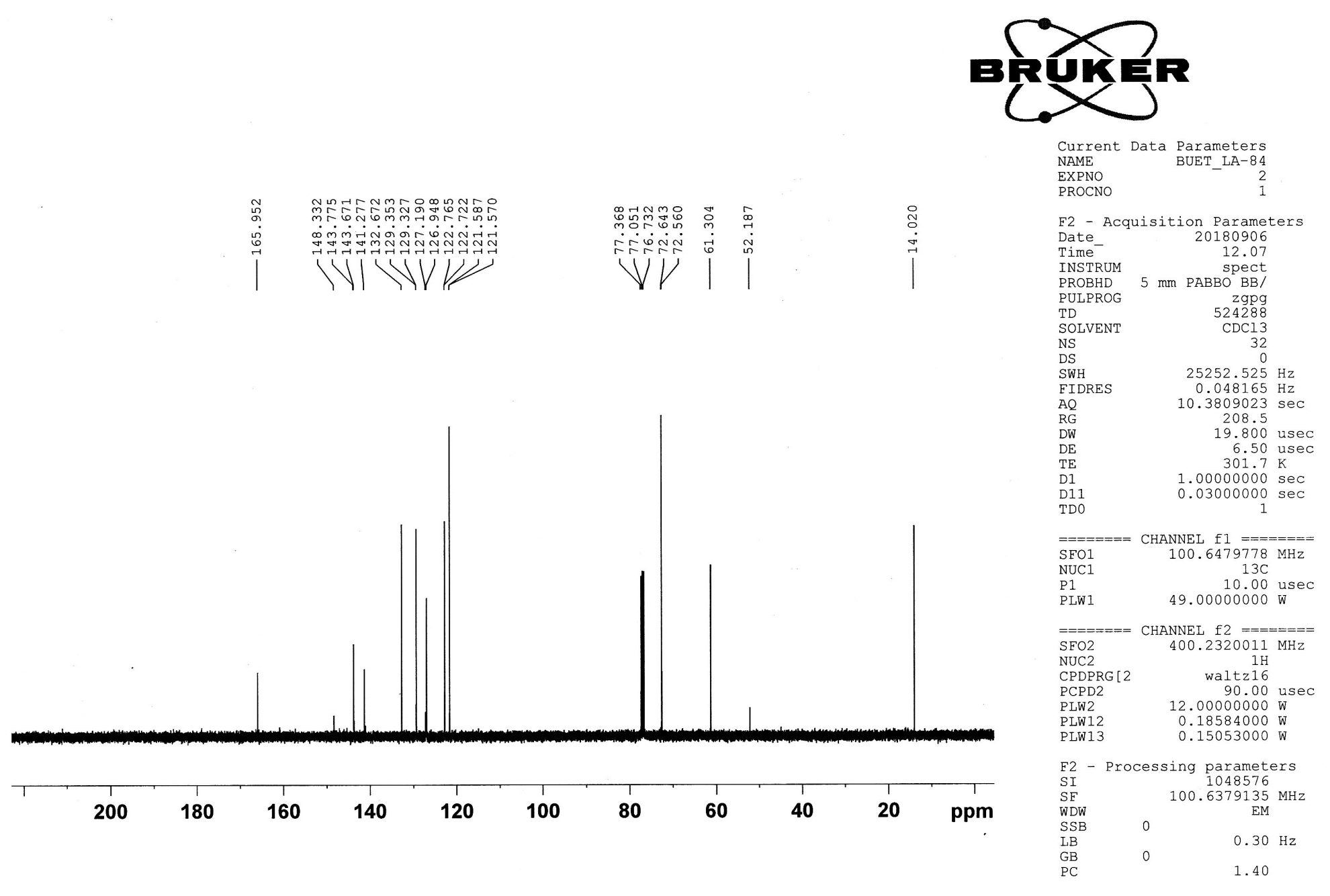

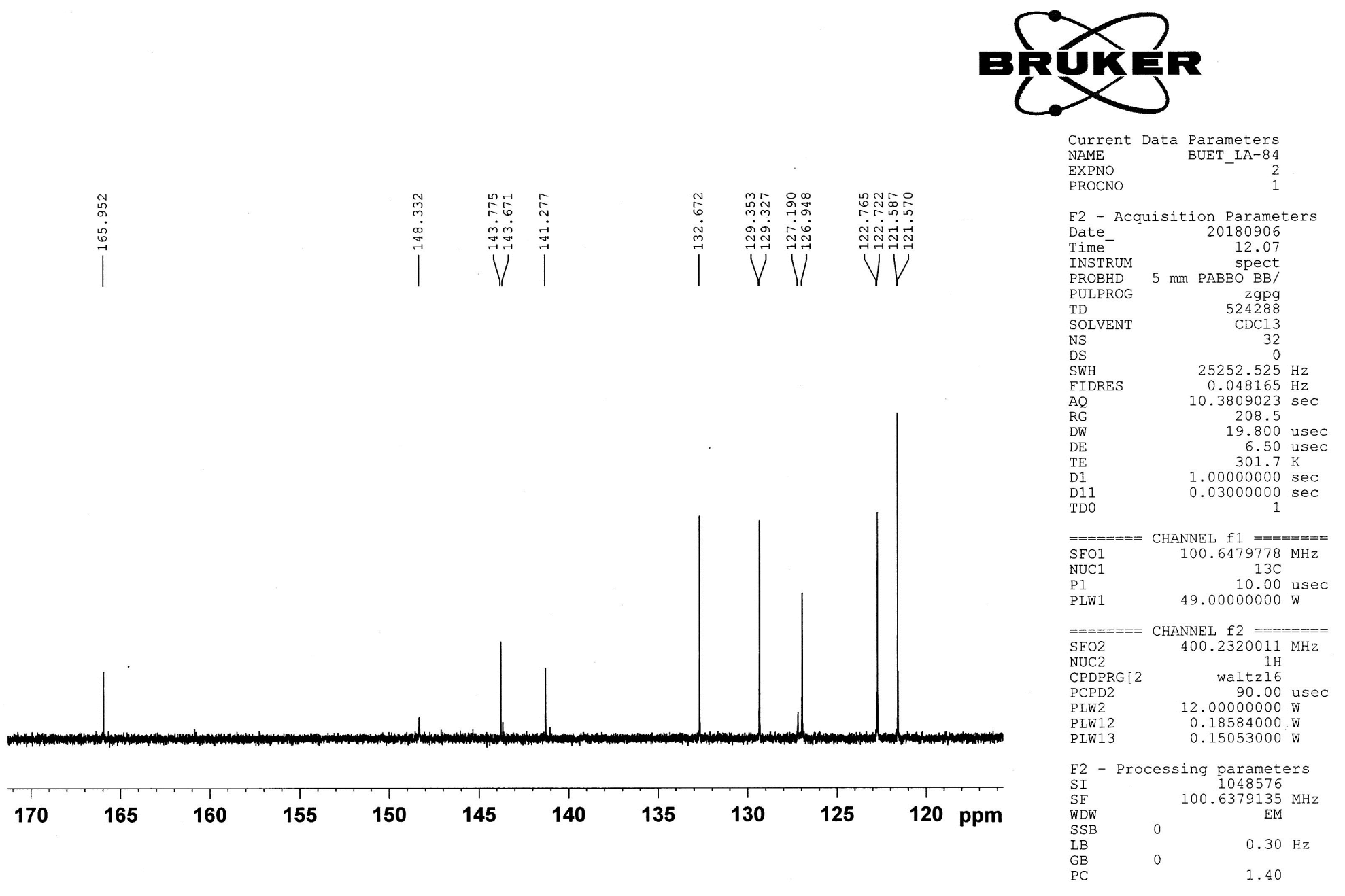


No. P/V Wavelength nm. Abs.

1 652.50 0.042

2 264.00 0.213

3 230.00 0.087

[Measurement Properties]

Wavelength Range (nm.): 200.00 to 800.00

Sampling Interval: 0.5

Auto Sampling Interval: Enabled

Scan Mode: Single

[Instrument Properties]

Instrument Type: UV-1800 Series

Measuring Mode: Absorbance

Slit Width: 1.0 nm

Light Source Change Wavelength: 340.0 nm

S/R Exchange: Normal

Threshold: 0.0010000

Points: 4


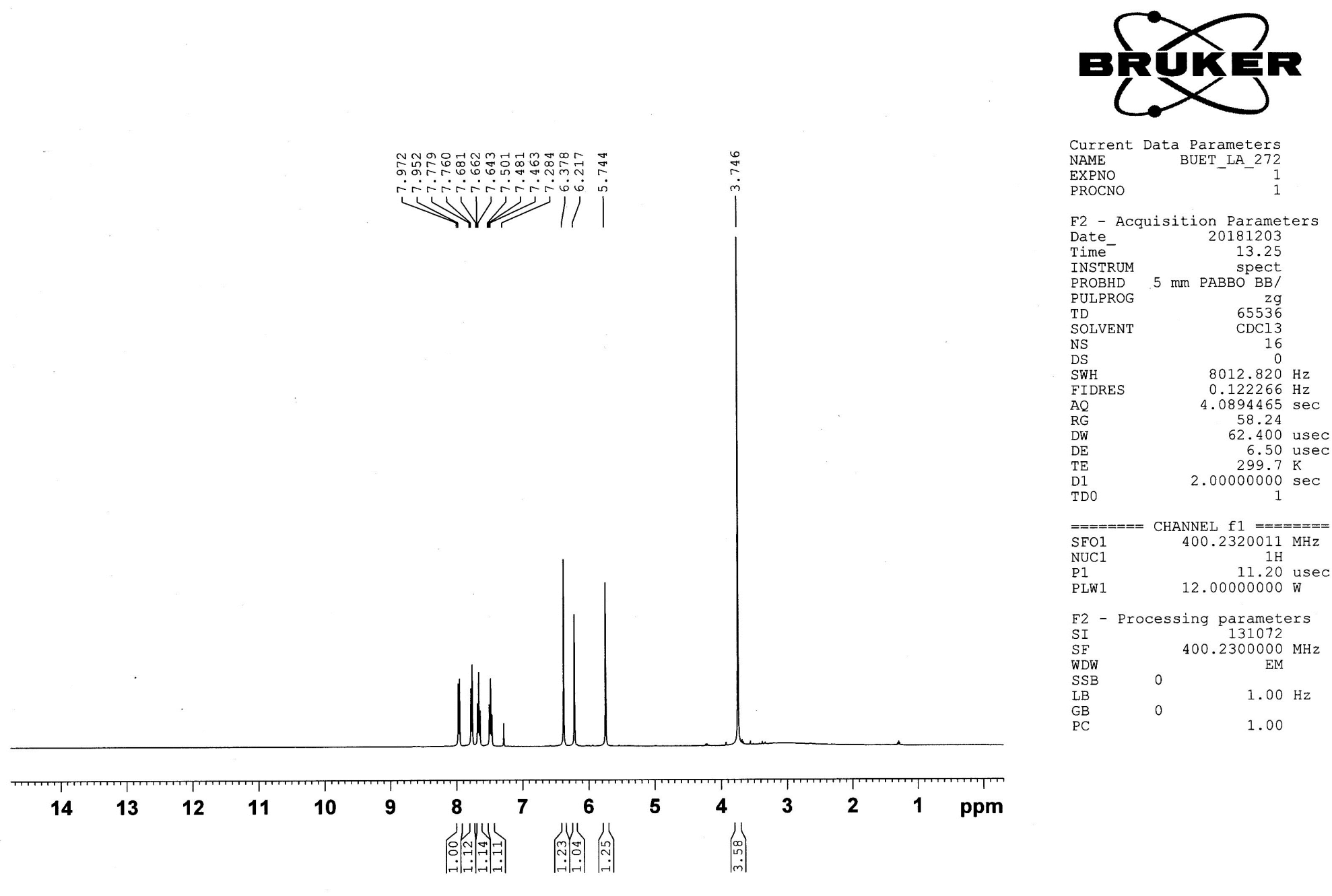
**Compound-**
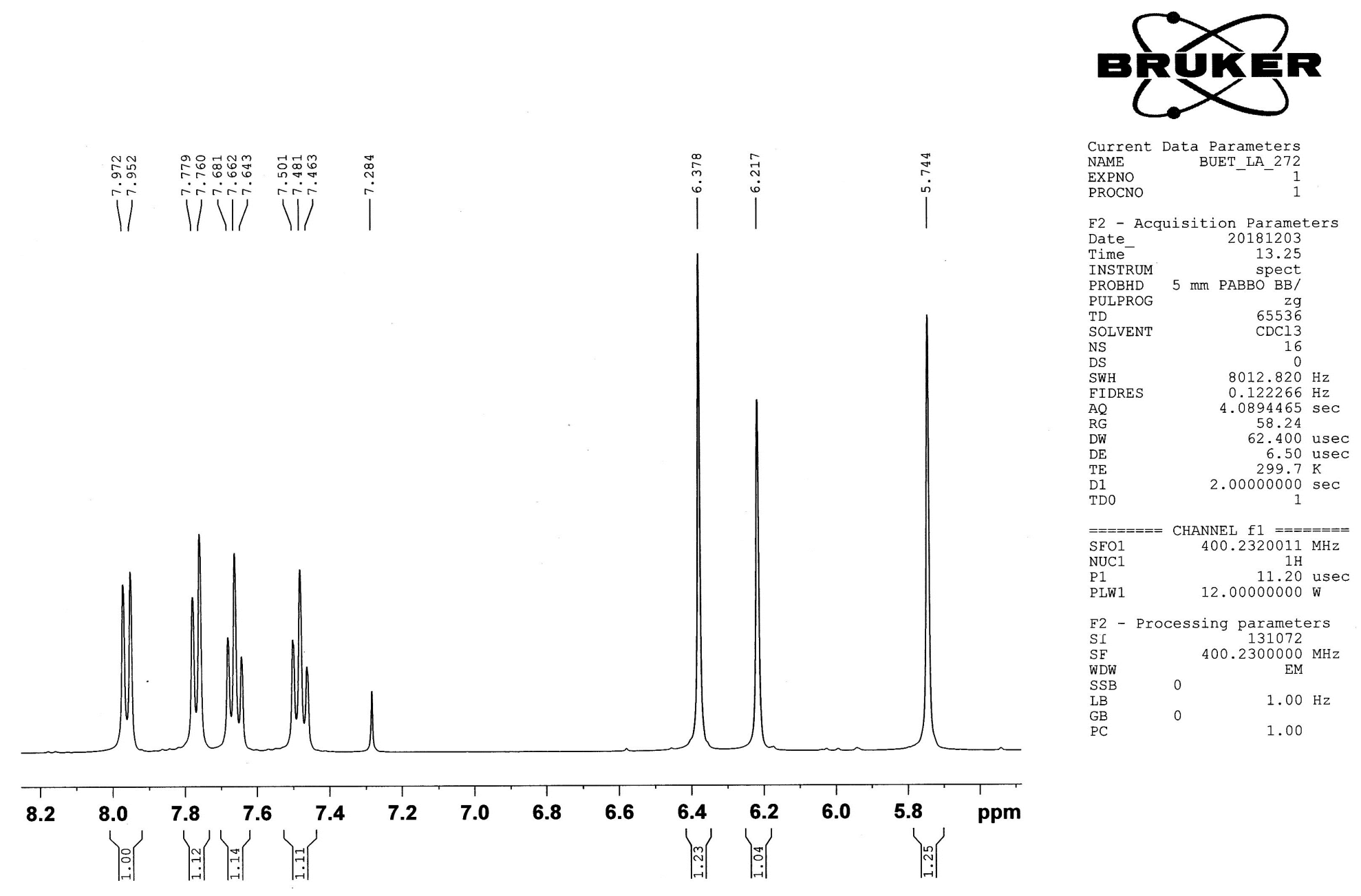
**10**

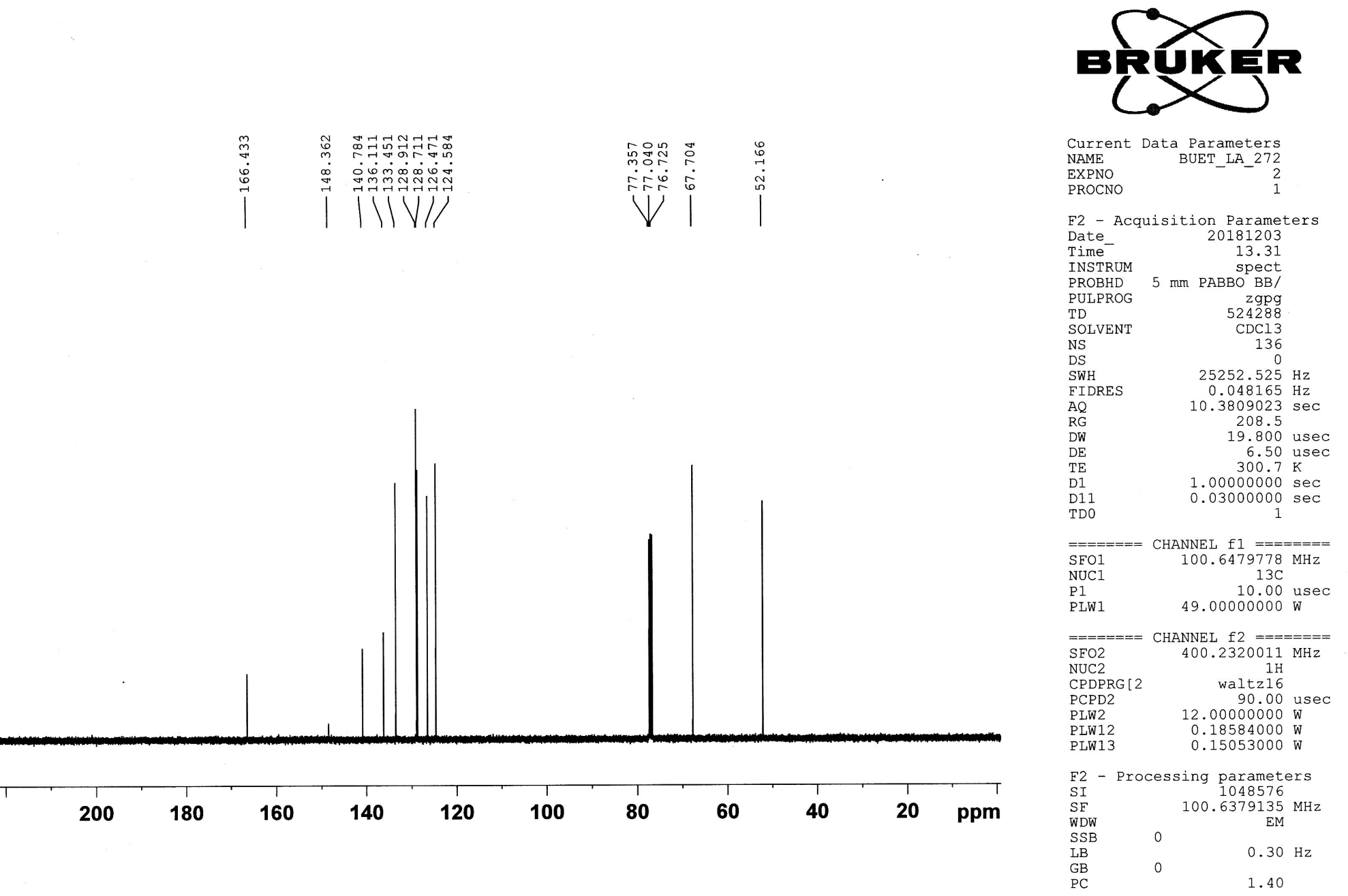

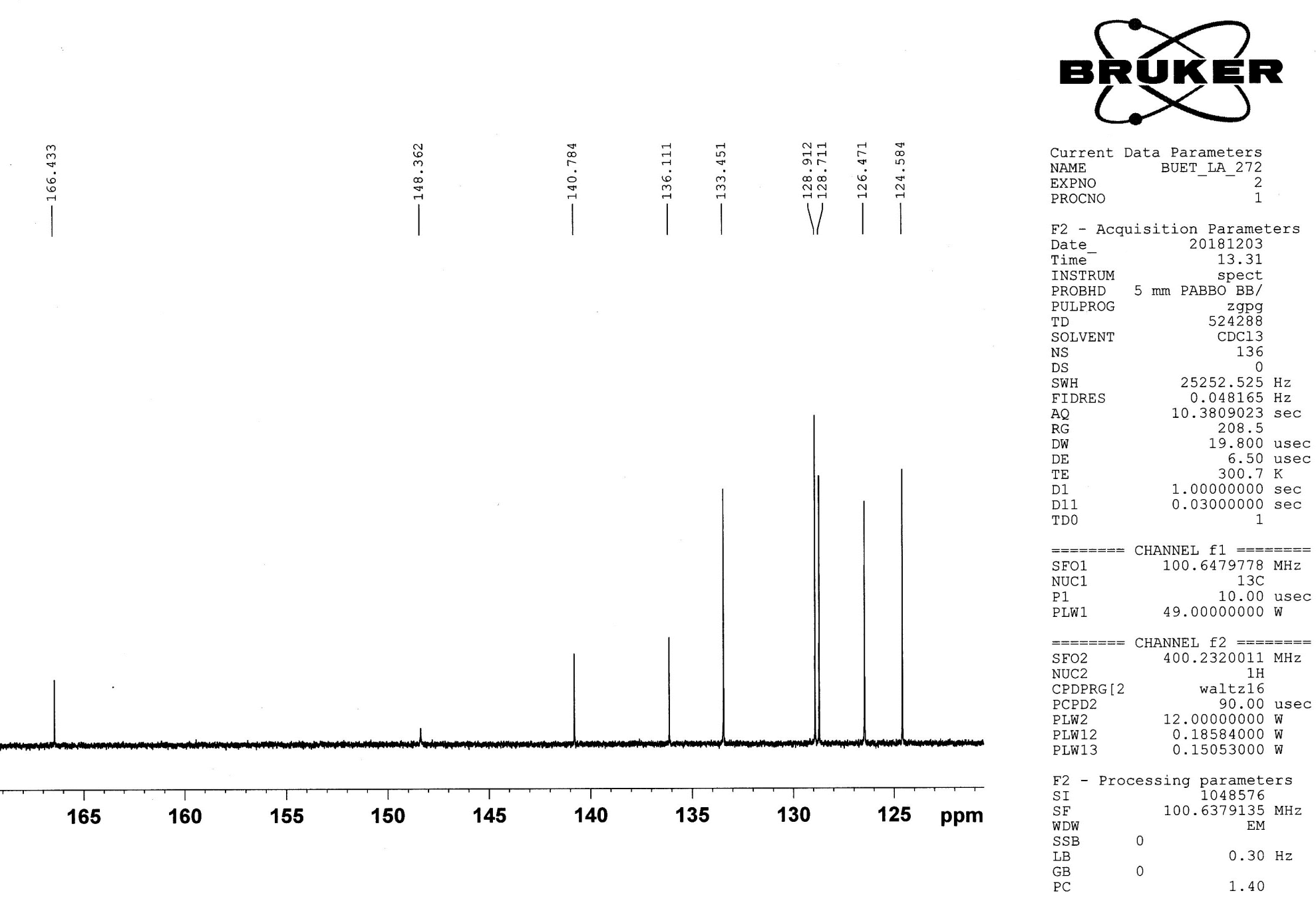

No. P/V Wavelength nm. Abs.

1 257.40 0.560

2 214.20 4.000

**Compound-**
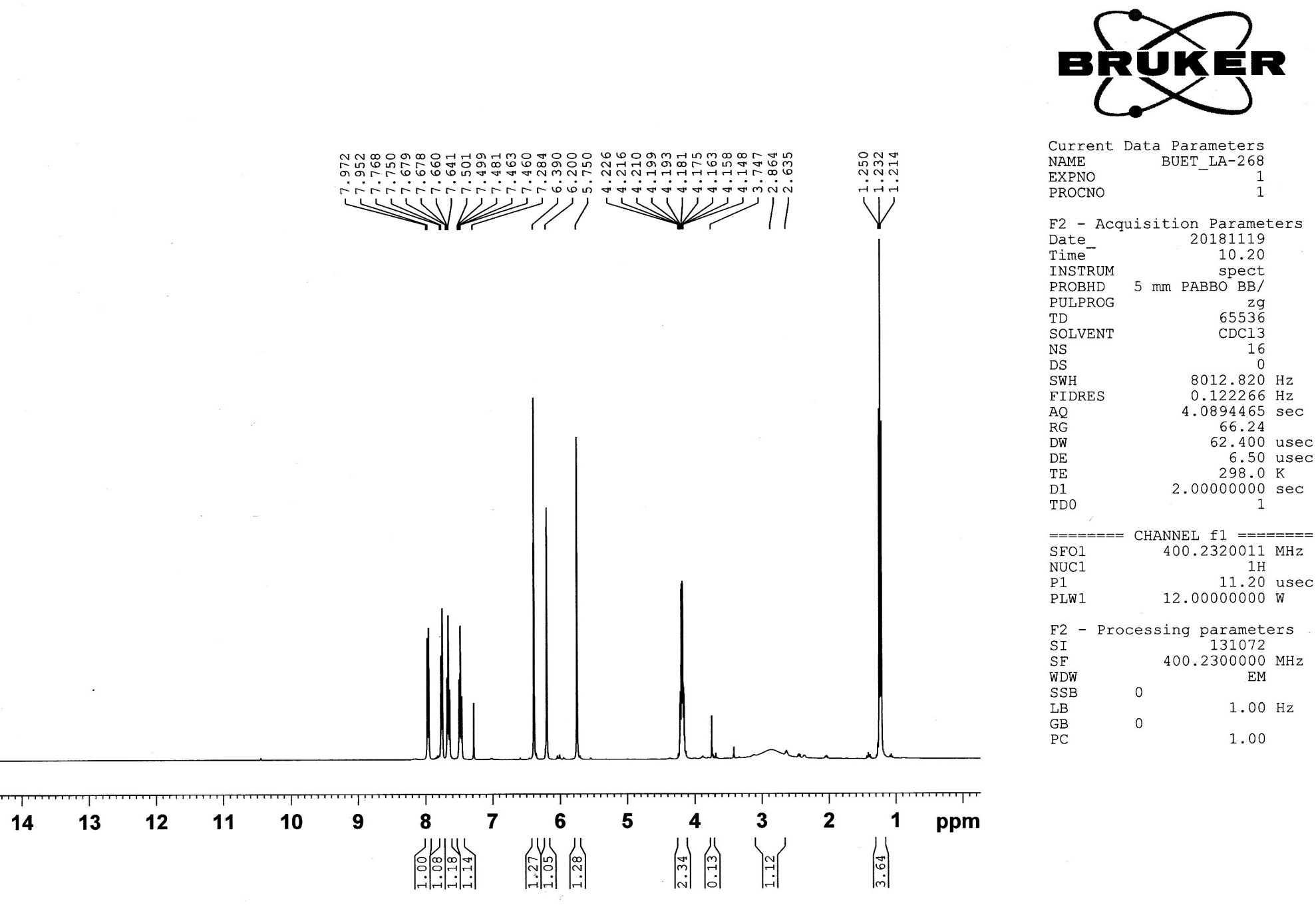

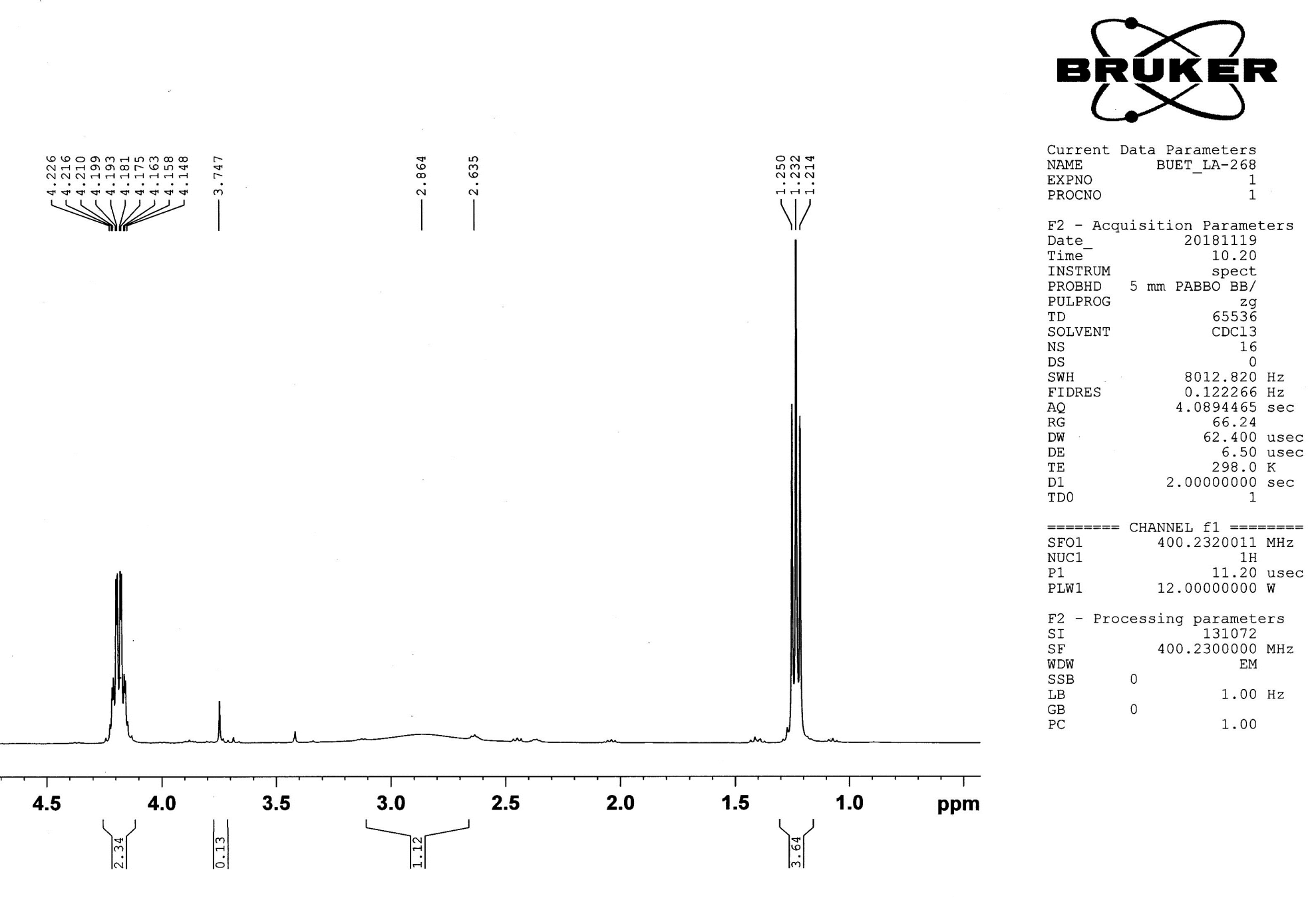
**11**

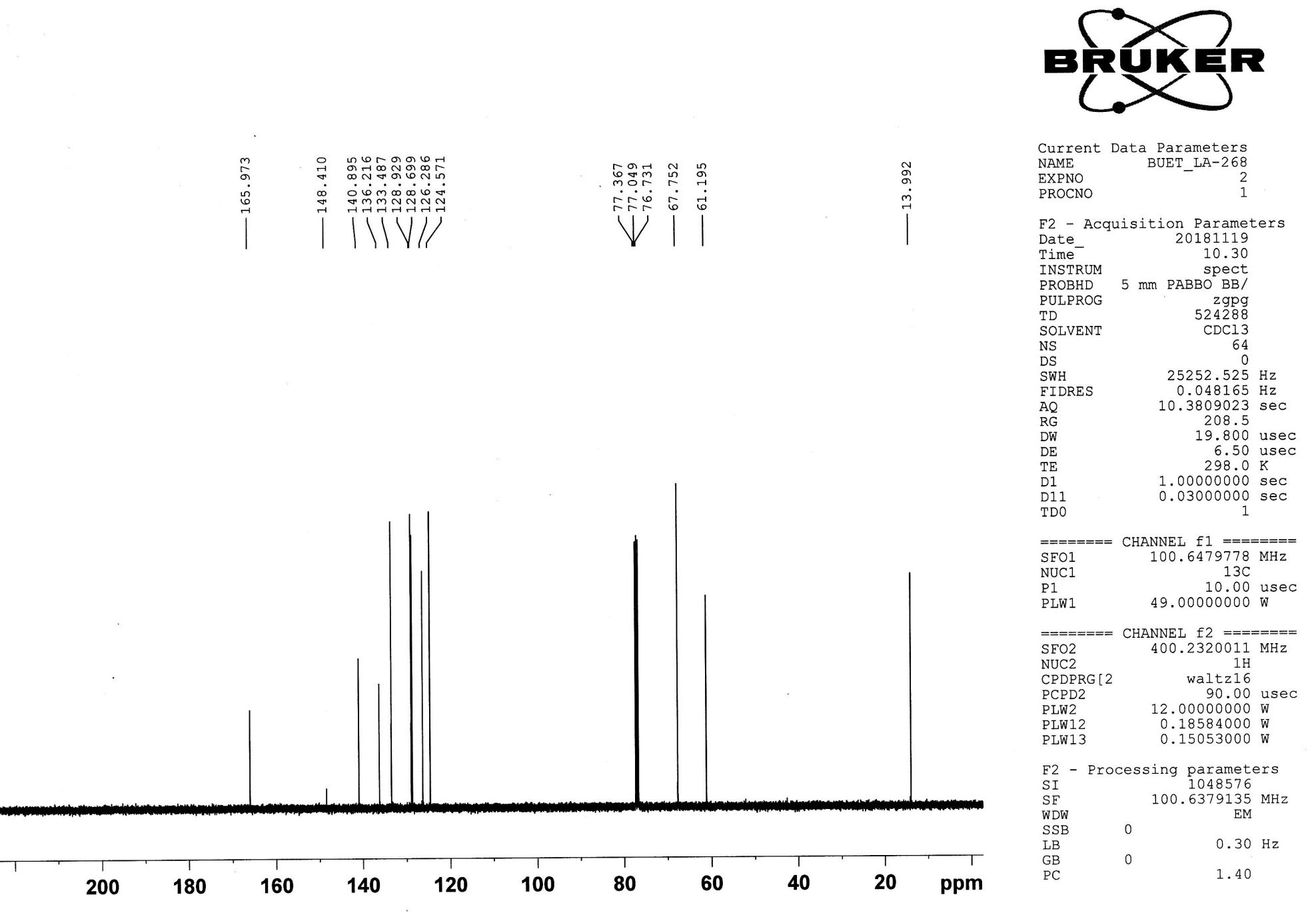

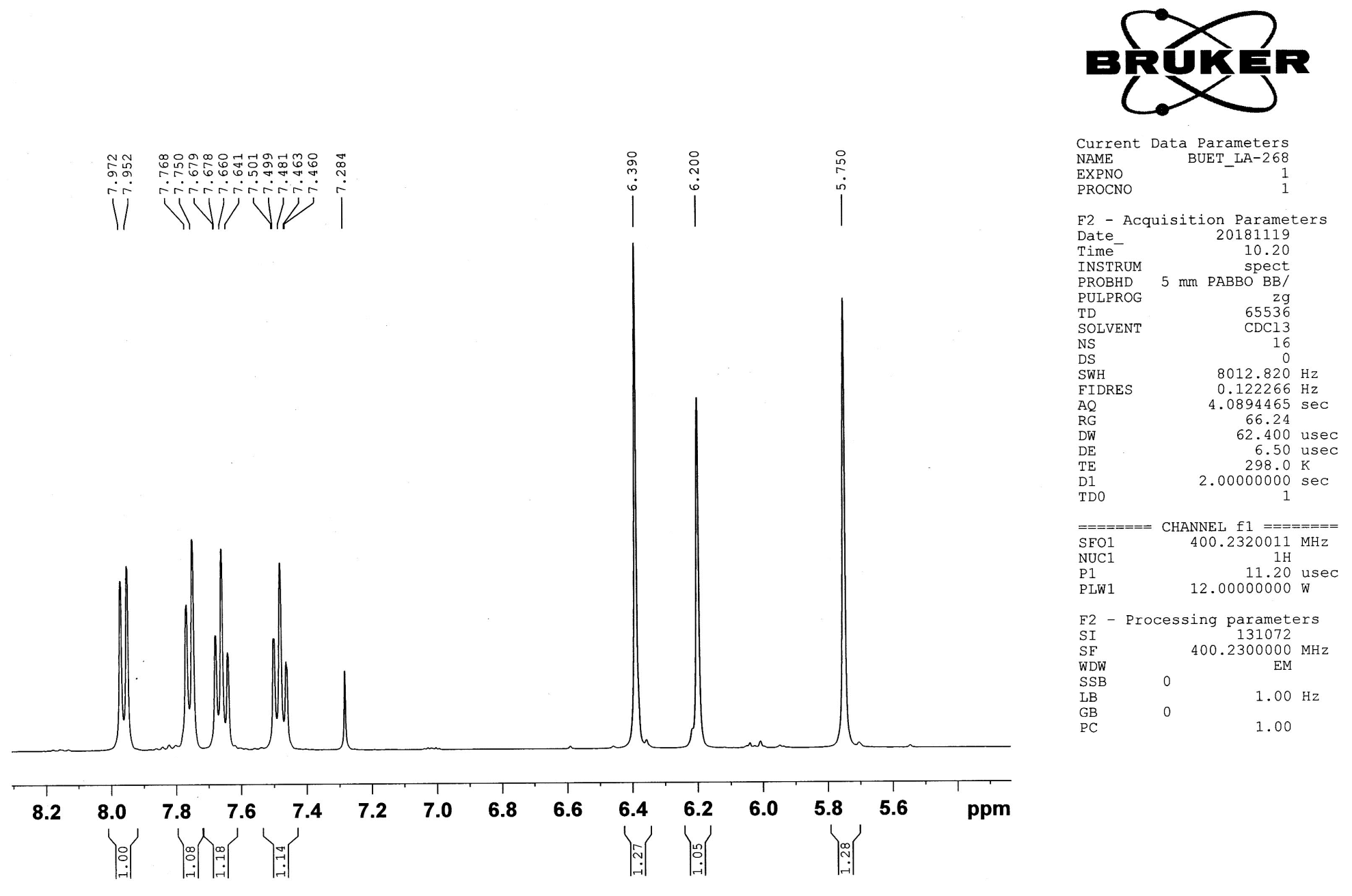

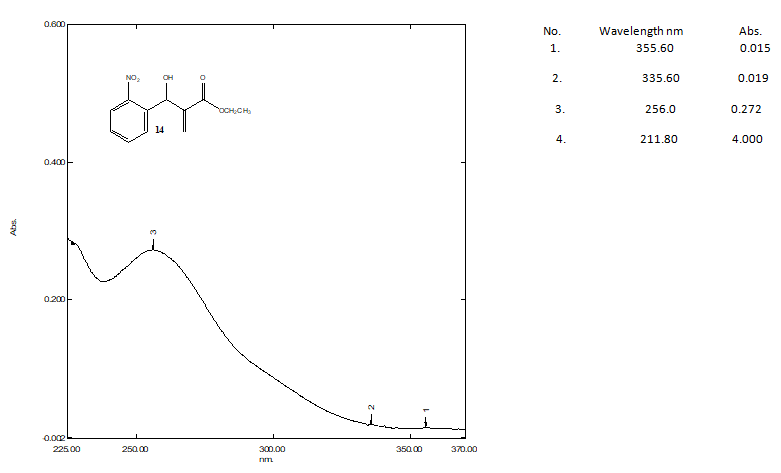


Figure-S2: Spetrum of 1H NMR, 13C NMR, UV and FTIR for 06, 07, 08, 9, 10 and 11

Table S2: Gibbs free energy for reactant and product

|  | Reactant | | | |  | Product | | | |
| --- | --- | --- | --- | --- | --- | --- | --- | --- | --- |
|  | ∆H | T | ∆S | ∆G |  | ∆H | T | ∆S | ∆G |
| No solvent | 9999.507 | 298 | 117.567 | -25033.38 |  | 9248.6393 | 298 | 112.343 | -24228.69 |
| Water | 5941.270 | 298 | 91.7800 | -21409.17 |  | 6371.8938 | 298 | 93.9495 | -21622.23 |
| MeOH | 7145.864 | 298 | 99.9045 | -22624.34 |  | 6405.6574 | 298 | 93.990 | -21603.37 |
| Dioxane | 9692.776 | 298 | 116.124 | -24912.17 |  | 9077.0237 | 298 | 111.773 | -24231.33 |
| DMSO | 9544.565 | 298 | 114.864 | -24683.72 |  | 9277.8187 | 298 | 114.716 | -24905.77 |
| t-Butanol | 9626.882 | 298 | 115.842 | -24893.33 |  | 9140.4449 | 298 | 112.043 | -24247.92 |
| DMF | 9620.984 | 298 | 115.723 | -24863.58 |  | 9127.3987 | 298 | 111.800 | -24189.01 |
| Toluene | 9708.704 | 298 | 116.105 | -24890.59 |  | 9248.5839 | 298 | 114.662 | -24960.5 |
| THF | 9635.694 | 298 | 114.960 | -24622.39 |  | 9262.4288 | 298 | 114.130 | -24751.3 |

Table S3: Heat of formation for reactant and product and Heat of reaction

| Solvents | Heat of formation(∆H_r_) for Reactant, eV | Heat of formation (∆H_p_) for Product, eV | Heat of reaction eV |
| --- | --- | --- | --- |
| No solvent | 325.83 | 272.33 | -53.5 |
| Water | 325.85 | 245.04 | -80.81 |
| MeOH | 326.55 | 246.24 | -80.31 |
| Dioxane | 326.83 | 250.21 | -76.62 |
| DMSO | 298.80 | 232.51 | -66.31 |
| t-Butanol | 289.82 | 225.73 | -64.09 |
| DMF | 284.12 | 222.33 | -61.79 |
| Toluene | 308.99 | 256.35 | -52.64 |
| THF | 292.90 | 241.25 | -51.65 |
